# Unravelling the relationship between exercise‐induced affective responses and daily physical activity in people with chronic diseases: Spillover or confounding effect?

**DOI:** 10.1111/bjhp.70097

**Published:** 2026-07-24

**Authors:** Layan Fessler, Dan Orsholits, Alexis Le Faucheur, Ryan E. Rhodes, Boris Cheval, Philippe Sarrazin

**Affiliations:** ^1^ Univ Grenoble Alpes, SENS Grenoble France; ^2^ Swiss Centre of Expertise in Life Course Research (Centre LIVES) Switzerland; ^3^ Univ Rennes, M2S – EA 7470 Rennes France; ^4^ School of Exercise Science, Physical and Health Education University of Victoria Victoria British Columbia Canada; ^5^ Univ Rennes, École Normale supérieure de Rennes, VIPS2 Rennes France; ^6^ Present address: University of Lille, CNRS, UMR 9193 ‐ SCALab ‐ Sciences Cognitives et Sciences Affectives Lille France

**Keywords:** effort, exercise hedonics, noncommunicable disease, spillover effect, timing

## Abstract

**Objectives:**

Exercise‐induced affective responses (ARs) may encourage people with chronic diseases (PCD) to engage in future physical activity (PA). This study examined whether AR timing during exercise predicts daily moderate‐to‐vigorous PA (MVPA) in PCD and explored two mediating pathways: the reflective affect processing pathway (via remembered pleasure, forecasted pleasure and affective attitudes) and the self‐efficacy pathway (via self‐efficacy towards exercise and PA).

**Design:**

Prospective correlational design.

**Methods:**

A total of 109 adults (M_age_ = 66.6 years, 79% women) with chronic diseases reported ARs and perceived exertion four times during an exercise session. Post‐exercise measures included remembered and forecasted pleasure, BMI, affective attitudes and self‐efficacy. Daily MVPA was measured using accelerometers over eight days. Total PA (light‐intensity PA and MVPA) was examined as a secondary outcome.

**Results:**

The last AR predicted daily MVPA (*β* = .24, *p* = .041), but this association became non‐significant after adjusting for confounders (BMI, perceived exertion). In contrast, the last AR predicted total PA independently of confounders (*β* = .30, *p* = .008). Exploratory mediation analyses partially supported the reflective affect processing pathway: ARs predicted affective attitudes via remembered and forecasted pleasure, though affective attitudes did not predict either PA outcome. The self‐efficacy pathway was supported for MVPA only, albeit with a modest indirect effect (*β* = .05).

**Conclusions:**

The last AR did not predict daily MVPA after adjusting for confounders, questioning a spillover effect from exercise sessions to MVPA. Secondary analyses suggest the last AR may predict total PA regardless of confounders, hinting at an intensity‐specific spillover. Findings should be interpreted cautiously given the limited sample size and the exploratory nature of secondary analyses.

**Statement of Contribution**

**What is already known on this subject?**

- Exercise‐induced affective responses (ARs) are hypothesized to predict subsequent daily physical activity (PA), suggesting a spillover effect from exercise to daily‐life activity
- The peak–end rule suggests that the most intense and last ARs of an exercise session significantly shape remembered and forecasted pleasure, which in turn may predict future PA behaviour.
- ARs may predict PA through two pathways: the reflective affect processing pathway (mediated by remembered pleasure, forecasted pleasure and affective attitudes) and the self‐efficacy pathway (mediated by self‐efficacy towards exercise and PA).

**What does this study add?**

- The positive association between the last AR and daily moderate‐to‐vigorous PA became non‐significant after adjusting for confounders (BMI and perceived exertion), though this should be interpreted cautiously given the limited sample size.
- Secondary analyses showed that the positive association between the last AR and total daily PA remained significant after adjusting for confounders, suggesting an intensity‐specific spillover effect, a finding that should be considered exploratory and interpreted with caution.
- While ARs were associated with both affective and cognitive processes, only the self‐efficacy pathway (not the reflective affect processing pathway) significantly predicted daily MVPA, underscoring the importance of cognitive mediators in PA behaviour in people with chronic diseases.

## INTRODUCTION

Chronic diseases, such as cardiovascular diseases, cancers and diabetes, account for 69% of all deaths worldwide and result in a cumulative global economic loss of US$7 trillion (Peter et al., 2022; World Health Organization, 2013, 2023). Regular physical activity (PA) is essential for both the prevention and management of these diseases (Bierbauer et al., 2020; Durstine et al., 2013; Kanaley et al., 2022; Spence et al., 2020). For example, cancer survivors who engage in the recommended level of 150 min of moderate‐to‐vigorous PA (MVPA) per week can reduce treatment side effects and improve their quality of life (Spence et al., 2020). However, many people with chronic diseases (PCD) do not meet these PA guidelines (Dagner et al., 2019; Goo et al., 2024). This gap highlights the need to better understand the determinants of PA engagement in this population.

In recent years, exercise‐induced affective responses (ARs) have emerged as a key predictor of PA engagement and maintenance, potentially offering insights beyond traditional socio‐cognitive theories that mostly focus on attitudes, intentions, or self‐efficacy (Brand & Ekkekakis, 2018; Cheval & Boisgontier, 2021; Ekkekakis, 2017; Ekkekakis et al., 2020; Stevens et al., 2020). According to the hedonic principle, actions are driven by the pursuit of pleasure and the avoidance of displeasure (Cabanac, 1992; Johnston, 2003; Williams, 2008). Therefore, experiencing positive ARs during exercise is expected to increase the likelihood of future PA engagement, as the hedonic value of the experience increases its motivational pull (Dunton et al., 2023; Rhodes et al., 2023; Williams, 2008). The Affect and Health Behaviour Framework (AHBF; Stevens et al., 2020; Williams & Evans, 2014)—a schematic structure used to label, organize and integrate affect‐related constructs to understand health‐related behaviour (Williams & Rhodes, 2023)—suggests that ARs influence PA through repetition and associative learning. These associations are encoded in memory through two distinct pathways: an automatic processing pathway (e.g., automatic affective associations and implicit attitudes) and a reflective processing pathway (e.g., remembered pleasure, forecasted pleasure and affective attitudes). In both pathways, ARs serve as the foundational roots of the motivational process leading to PA behaviour (Dunton et al., 2023; Stevens et al., 2020).

Beyond these two affect processing pathways, Bandura's self‐efficacy theory (Bandura, 1977) identifies affective and physiological states as a foundational source of self‐efficacy. Accordingly, ARs experienced during exercise may shape self‐efficacy beliefs, which in turn predict PA behaviour. Thus, experiencing negative ARs during PA may discourage future engagement and hinder the achievement of recommended PA levels. This effect is particularly relevant for PCD, who may experience negative ARs more frequently due to factors such as chronic pain, discomfort, or exercise‐related anxiety (e.g., Aydemir et al., 2022; Goubran et al., 2025). Additionally, self‐efficacy has been identified as an important determinant of PA engagement in PCD (e.g., Selzler et al., 2020), further supporting the relevance of this pathway in the present population.

**FIGURE 1:**
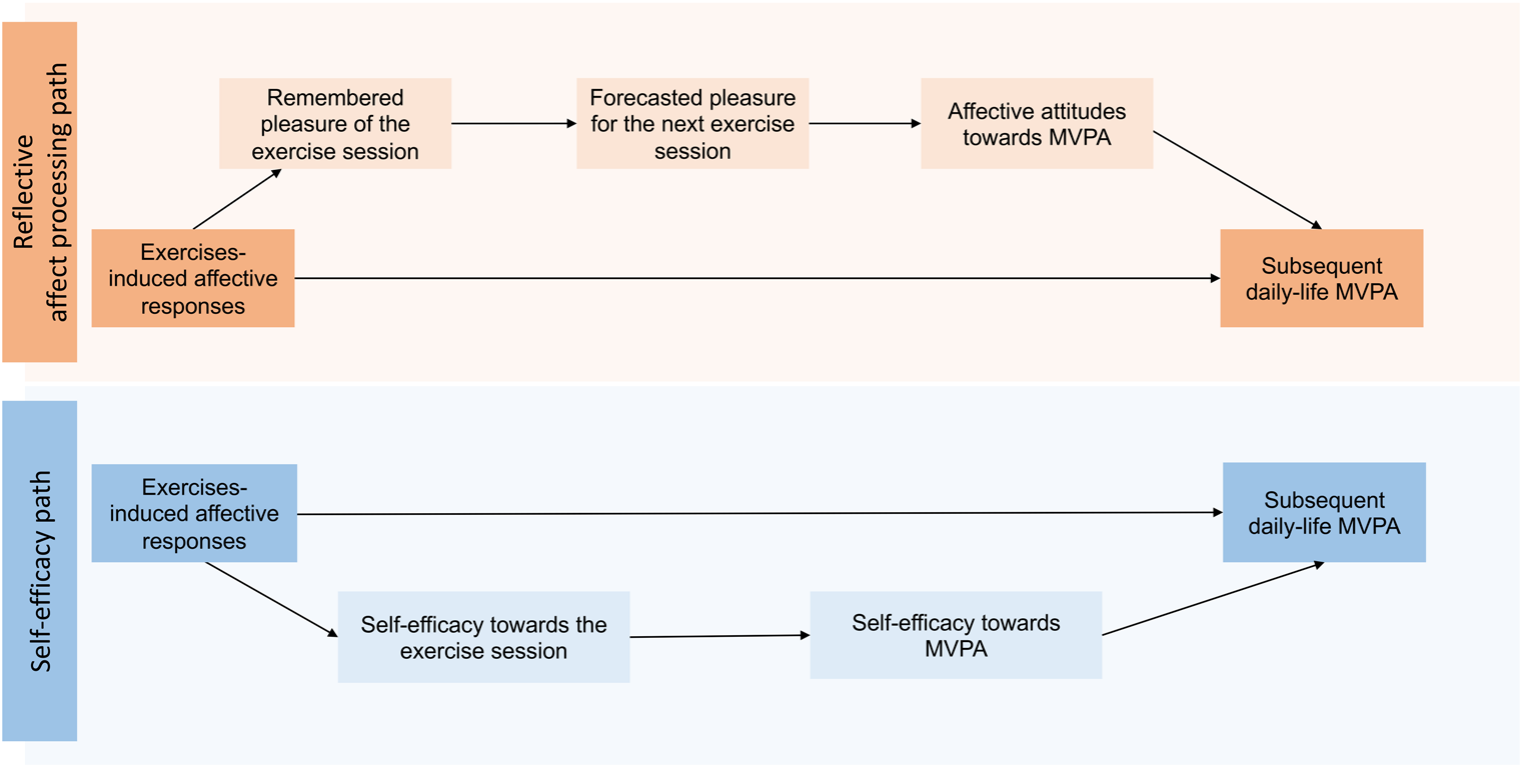
Reflective affect processing and self‐efficacy pathways. MVPA, moderate‐to‐vigorous physical activity. The reflective affect processing pathway is modelled within the AHBF (Stevens et al., 2020; Williams & Evans, 2014). The self‐efficacy pathway is grounded in Bandura's self‐efficacy theory (Bandura, 1977).

The present study examined two pathways through which ARs may influence PA behaviour (Figure 1): the reflective affect processing pathway, as modelled in the AHBF and a self‐efficacy pathway, grounded in Bandura's self‐efficacy theory (Bandura, 1977). Although the self‐efficacy pathway is not formally incorporated as a distinct processing pathway within the AHBF, Stevens et al. (2020) explicitly call for research examining how affect‐related and traditional cognitive factors—including self‐efficacy—interrelate to influence PA behaviour, providing theoretical justification for its inclusion in the present study. The automatic processing pathway—through affective associations and implicit attitudes—was beyond the scope of the present study.

A growing body of evidence shows that positive exercise‐induced ARs are associated with higher levels of PA across diverse populations, including healthy active adolescents (Schneider et al., 2009), healthy active and inactive adults (Kwan & Bryan, 2010; Williams et al., 2012), and overweight or obese inactive adults (Liao et al., 2017; Williams et al., 2008, 2016). For example, in a study with 37 overweight, physically inactive adults, Williams et al. (2008) found that positive ARs during a moderate‐intensity treadmill session predicted higher self‐reported PA at 6 and 12 months. Similarly, Kwan and Bryan (2010) found that increases in positive affect and decreases in fatigue during a single exercise session predicted more frequent voluntary exercise participation at 3‐month follow‐up in a sample of adults varying in baseline activity level. Additionally, using an ecological momentary assessment, Liao et al. (2017) reported that fewer negative and higher positive ARs during PA were associated with higher accelerometer‐based daily MVPA after 12 months in 82 overweight, physically inactive adults. Taken together, these findings suggest that positive exercise‐induced ARs may lead to a ‘spillover effect’ on subsequent PA in both active and inactive populations. The contextual spillover effect has been defined as a behaviour (e.g., PA) conducted in context 1 (e.g., an exercise session) affecting the probability of the same behaviour being conducted in context 2 (e.g., daily‐life PA; Nilsson et al., 2017). In other words, the ARs experienced during an exercise session are expected to influence the likelihood of engaging in more PA in daily life.

Beyond the influence of the average AR on subsequent PA behaviours, the ‘peak–end rule’ suggests that the most intense (‘peak’) and last (‘end’) affective moments of an exercise session may significantly shape remembered and forecasted pleasure, thereby influencing future PA participation (Fredrickson, 2000; Kahneman et al., 1993). For example, a recent study showed that healthy adults who experienced more positive ‘peak’ and ‘end’ affective responses during aerobic and resistance training sessions remembered the sessions as more pleasurable and anticipated the next session as more pleasant (Bastos et al., 2025). In turn, both remembered and forecasted pleasure were found to positively predict exercise frequency after the intervention. With regard to PCD, Fessler et al. (2024) conducted a preliminary study in seven Parkinson's disease patients, showing that adding nine minutes of low‐intensity exercise at the end of weekly sessions improved affective attitudes towards PA. However, this shift in attitudes did not translate into increases in subsequent daily‐life PA levels. While the peak–end rule primarily informs how affective experiences are cognitively represented, the hedonic principle suggests that ARs—or their salient representations—can directly influence future behavioural repetition (Cabanac, 1992; Johnston, 2003; Williams, 2008). From this integrative perspective, ARs experienced at particularly salient moments—such as the end of an exercise session—are more readily accessible in memory and may therefore serve as stronger inputs to the hedonic appraisal process that drives future behavioural repetition, while also shaping remembered and forecasted pleasure through the reflective affect processing pathway (Kahneman, 2000).

Although these studies provide initial evidence for the potential role of AR in the regulation of PA, they also present mixed findings and several limitations. First, some studies have found that the association between ARs and PA may not be significant once potential confounders, such as perceived exertion, have been considered (Williams et al., 2008, 2012). This calls into question the validity of a potential direct effect between AR and subsequent PA. Second, the majority of studies—with a few exceptions, such as Liao et al. (2017)—have measured ARs following a graded submaximal exercise test in a laboratory setting (Kwan & Bryan, 2010; Schneider et al., 2009; Williams et al., 2008, 2012). This raises the question of whether ARs experienced during real‐world exercise are related to subsequent daily PA. Third, studies have typically examined the reflective affect processing and self‐efficacy pathways separately rather than comparing their respective contributions (e.g., Bastos et al., 2025; Rhodes et al., 2023; Warner et al., 2014). This gap limits our understanding of how these pathways may differentially predict PA behaviour in PCD. Finally, although ARs have the potential to predict PA in PCD, most research has focused on healthy populations. This focus excludes common chronic diseases, such as cardiovascular diseases, cancer, chronic respiratory diseases and diabetes (World Health Organization, 2023), limiting our understanding of how ARs may influence PA in these groups. In summary, the current evidence on the impact of the last AR of an exercise session on future PA participation remains inconclusive at best. To address these gaps, the present study investigated whether ARs assessed at specific points during an exercise session could predict subsequent daily MVPA in PCD. Using a prospective, ecological design, participants reported their ARs and perceived exertion at four time points during the session, followed by assessments of remembered and forecasted pleasure. As the peak AR can occur at any time during an exercise session (Fredrickson, 2000), capturing it through self‐reported measures is inherently challenging. Discrete measurements at arbitrary time points may fail to identify the true peak AR. In contrast, the end of an exercise session provides a consistent and reliable reference point. Therefore, we focused on the ‘end’ AR, representing the final affective response of the session.

Based on the hedonic principle (Cabanac, 1992; Johnston, 2003; Williams, 2008) and previous empirical studies (Liao et al., 2017; Rhodes & Kates, 2015; Williams et al., 2016), we hypothesised that the average AR through an exercise session would positively predict subsequent daily MVPA (*H*_1_: spillover effect). Drawing on the hedonic principle and informed by the peak–end rule heuristic—which suggests that end affect carries disproportionate weight in the overall representation of an affective experience (Fredrickson, 2000; Kahneman et al., 1993)—we hypothesized that ARs experienced at the end of an exercise session would show a stronger association with subsequent daily MVPA than ARs reported earlier in the session (*H*_2_: end effect). This hypothesis does not assume that the peak–end rule directly predicts behaviour, but rather that the differential salience of end affect makes it a stronger hedonic input to the process driving behavioural repetition. Additionally, we explored whether this spillover effect (both average AR and each AR) remained significant after adjusting for potential confounders in order to determine whether the effect reflects a true spillover rather than a confounding influence (exploratory analysis 1 [*EA*_1_]). Further exploratory analyses investigated the two potential pathways through which ARs may predict MVPA. First, we examined the reflective affect processing pathway, mediated serially by remembered pleasure, forecasted pleasure and affective attitudes (*EA*_2_). Second, drawing on Bandura's (1977) theory that affective states influence self‐efficacy beliefs, we explored a self‐efficacy pathway in which ARs may predict MVPA through a serial mediation via self‐efficacy towards exercise and self‐efficacy towards PA (*EA*_3_, see Figure 1). Finally, secondary analyses were conducted using total PA—encompassing light‐intensity PA and MVPA—as the dependent variable to provide a more comprehensive evaluation of potential spillover effects across the full spectrum of PA intensities.

## METHODS

### Study design and power analysis

A prospective ecological design was used, in which participants engaged in an exercise session provided by their usual exercise and health structure. Their daily PA levels were then measured over a period of eight days. To estimate the minimum sample size required to test the main study hypotheses (*H*_1_ and *H*_2_), we conducted a priori power analyses using G*Power (v. 3.1; Faul et al., 2009). The calculation was based on the smallest effect sizes reported in prior similar studies involving adults (Williams et al., 2008, 2012). For a linear regression with one predictor (average AR and ARs at one time point), an alpha level of 0.05 and a desired power of 0.90, a minimum of 178 participants was required to detect an effect size of *f* ^2^ = 0.06/*r =* .24 (Williams et al., 2012) (Data S1, Figure S1). However, practical constraints related to resources and time limited recruitment to 116 participants (Lakens, 2022), of whom 109 met the inclusion criteria and were included in the final analyses (Table 1). No power analysis was conducted for exploratory analyses (*EA*_1–3_), as their purpose was to uncover patterns, generate insights and lay the groundwork for subsequent hypothesis‐driven research, rather than to provide conclusive evidence (Haile, 2023).

**TABLE 1:** Descriptive characteristics.

*N* = 109	*N* (*%*)	Missing (*n*, %)
Age (year*s*, mean, *SD*)	66.56 (12.55)	7 (4.42)
< 50 (years)	7 (6.86)	
50–59 (years)	12 (11.76)	
60–69 (years)	44 (43.14)	
70–79 (years)	24 (23.53)	
> 80 (years)	15 (14.71)	
Gender		7 (4.42)
Woman	81 (79.41)	
Man	21 (20.58)	
Body mass index (kg/m^2^)	26.46 (5.84)	7 (4.42)
Underweight (< 18.5)	4 (3.92)	
Normal (18.5–24.9)	40 (39.22)	
Overweight (25–29.9)	35 (34.31)	
Obesity (> 30)	23 (22.55)	
Chronic disease (*n*, frequency)		11 (10.09)
Cardiovascular disease	9 (9.18)	
Hypertension	20 (20.41)	
Hypercholesterolemia	12 (12.24)	
Stroke	1 (1.02)	
Diabetes	11 (11.22)	
COPD	7 (7.14)	
Cancer	15 (15.31)	
Stomach ulcer	0 (0)	
Gastroduodenal ulcer	3 (3.06)	
Parkinson's disease	3 (3.06)	
Alzheimer's disease	0 (0)	
Dementia	1 (1.02)	
Senility	1 (1.02)	
Affective disorders	15 (15.31)	
Scoliosis	0 (0)	
Cataract	8 (8.16)	
Rheumatoid arthritis	7 (7.14)	
Osteoarthritis	39 (39.80)	
Other	25 (25.51)	
None	14 (14.29)	
Multimorbidity		11 (10.09)
Yes	52 (53.06)	
No	46 (46.934)	
Financial comfort		23 (21.10)
Very comfortable	4 (4.65)	
Fairly comfortable	36 (41.86)	
With some difficulties	44 (51.16)	
With great difficulties	2 (2.33)	
Education		23 (21.10)
Primary	85 (98.84)	
Secondary	77 (89.53)	
Tertiary	77 (89.53)	

*N* = 109	Mean (SD)	Missing (*n*, %)
Past self‐reported PA (hour/week)		22 (20.18)
GPAQ transport	8.79 (19.62)	
GPAQ moderate	7.50 (18.78)	
GPAQ vigorous	2.44 (4.61)	
GPAQ MVPA	9.94 (20.77)	
GPAQ sedentary	5.50 (2.73)	
Accelerometer‐based PA levels (min/day)		29 (26.61)
Light activity	102.34 (46.41)	
Moderate activity	37.51 (23.06)	
Vigorous activity	1.88 (3.86)	
MVPA	39.38 (24.48)	
Total PA	141.73 (60.35)	
Meeting the PA‐recommended level (*n*, %)	58 (72.50)	
Exercise and health programme membership (months)	74.66 (110.48)	22 (20.18)
Intention [1–7]	5.53 (1.65)	17 (15.60)
Affective attitudes [1–7]	5.26 (1.45)	23 (21.10)
Instrumental attitudes [1–7]	6.07 (1.67)	23 (21.10)
Self‐efficacy towards PA [1–7]	5.32 (1.68)	23 (21.10)

*Note*: Descriptice statistics were carried out on nonimputed data.

Abbreviations: OPD, chronic obstructive pulmonary disease; GPAQ, Global Physical Activity Questionnaire; MVPA, moderate‐to‐vigorous physical activity; PA, physical activity.

To determine the range of effect sizes detectable with our sample of 109 participants, sensitivity analyses were conducted using G*Power. With an alpha level of .05 and statistical power ranging from 33%—the lower threshold for insufficient power (Simonsohn et al., 2014)—to 90%, the sensitivity analysis showed that the study could detect effect sizes of *f* ^2^ = .02 (*r =* .14) at 33% power and *f* ^2^ = .10 (*r* = .31) at 90% power (Figure S2). The sensitivity power plot (Figure S2) showed that the study had 73% power to detect the minimum effect size observed in the literature for *H*_1_ and *H*_2_ (*f* ^2^ = .06), which falls below the conventional 80% threshold recommended by Cohen (1988). The study was therefore adequately powered to detect effect sizes of *f* ^2^ = .10 or above, but underpowered to reliably detect the smallest effect reported in prior research. Null findings, particularly for effect sizes below *f* ^2^ = .10, should be interpreted with caution, as they may reflect insufficient statistical power (i.e., an elevated risk of Type II error) rather than a true absence of significant association. Sensitive power analyses for exploratory analyses are detailed in Data S1.

### Participants

The study was approved by the French National Ethics Committee for Research in Sport Sciences (reference number: IRB00012476‐2022‐08‐03‐159). Participants were recruited from community‐based, structured exercise and health programmes for PCD in France—known as ‘adapted PA associations’—using flyers, emails and word of mouth. Participants were recruited regardless of how long they had been enrolled in the exercise and health programmes. This recruitment strategy was primarily driven by feasibility considerations, including access to the target population, time constraints and available resources, given the known challenges of recruiting large samples of PCD (Austin et al., 2025). Participants were included in the study if they met the following criteria: (a) age over 18 years; (b) had declared being exempt from any medical condition that would prohibit unsupervised PA; (c) were part of a rehabilitation programme or were enrolled in a sports club or association that includes at least one session of PA per week; (d) had declared at least one chronic disease or being enrolled in exercise and health programmes for specific groups requiring special precautions (i.e., vulnerable individuals); (e) without a history of serious psychiatric, neurological or mental disorders, or taking psychotropic or illicit drugs at the time of the study; and (f) were able to fully understand spoken and written French. Compliance with the inclusion criteria was determined with the help of each adapted PA association's team. Participants were recruited regardless of how long they had been enrolled in the exercise and health programmes. All participants provided written informed consent prior to participation and received no compensation.

### Procedure

**FIGURE 2:**
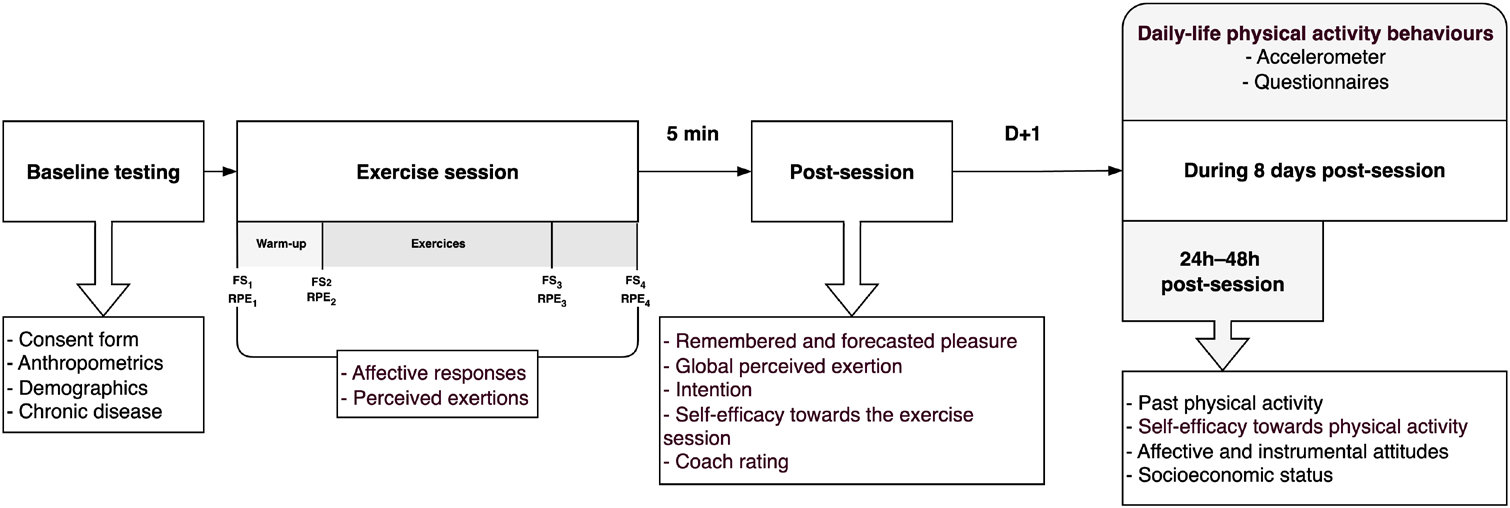
Overview of the study procedure. D + 1, the day after the exercise session; FS, Feeling Scale; PA, physical activity; post‐session, after the exercise session; RPE, rating of perceived exertion. FS and RPE were measured four times during an exercise session: at the start of the warm‐up (FS_1_, RPE_1_), at the start of the first exercise (FS_2_, RPE_2_), at the start of the last exercise (FS_3_, RPE_3_) and at the last minute of the session (FS_4_, RPE_4_).

Figure 2 provides an overview of the study protocol, while detailed characteristics of the exercise session are presented in Data S2. After enrolment, participants completed a questionnaire collecting demographic and health‐related information, including date of birth, height, weight, gender, duration of enrolment in the exercise and health programme (in months) and the presence of any chronic diseases or health conditions. The list of chronic diseases was adapted from the Survey of Health, Ageing and Retirement in Europe (SHARE; Börsch‐Supan et al., 2013). Participants were classified as having multimorbidity if they had two or more chronic diseases (Duggal et al., 2019). The participants then took part in their usual exercise session, supervised by a certified coach from the exercise and health programme. To maintain ecological validity, coach supervision was not standardized across exercise modalities. Instead, they were asked to lead their sessions as they normally would.

### Measures

#### Immediate affective responses

ARs were measured at four time points during each exercise session: the start of the warm‐up, the start of the first exercise, the start of the last exercise and the final minute of the session—that is, the moment immediately preceding the coach's formal termination of the session. All four measurements were taken during active exercise phases, ensuring that the last measurement captured the affective experience during ongoing exertion rather than during post‐exercise recovery. Due to variability in session duration across programmes, time points were determined by session structure rather than fixed intervals. Coaches briefed the experimenter beforehand on the session structure to facilitate accurate timing. All measurements were taken during active exercise phases, in line with recommendations to measure core affect in vivo and to avoid affective rebound effects that can occur rapidly following the cessation of effort (Andrade et al., 2022; Ekkekakis et al., 2011; Henriques et al., 2023). ARs were assessed by presenting participants with a printed copy of the Feeling Scale (FS; Hardy & Rejeski, 1989) and recording their verbal response. Participants were asked: ‘How do you feel right now, at this moment?’ and responded on an 11‐point scale ranging from −5 (*very bad*) to +5 (*very good*), with anchors at odd numbers and zero. Before the start of the exercise session, participants were familiarized with the scale by the experimenter. All sessions were conducted in small groups of three to six participants. To minimize peer influence, participants were asked either to point to their response on the scale or to speak quietly so that only the experimenter could hear their answer.

#### Reflective affect processing

Remembered pleasure and forecasted pleasure were measured five minutes after the end of the session using single items adapted from Kwan et al. (2017). Remembered pleasure was assessed with: ‘How would you qualify the time you spent during today's exercise session?’ Forecasted pleasure was assessed with: ‘How would you qualify the time you expect to spend during your next exercise session?’ Both items used a scale from −5 (*very unpleasant*) to +5 (*very pleasant*). To minimize common method variance between the FS and these measures (Podsakoff et al., 2003, 2012), the FS was presented vertically, while the remembered and forecasted pleasure scales were displayed horizontally (Hutchinson et al., 2023).

Affective attitude towards PA was assessed 24–48 h after the exercise session using two items (Phipps et al., 2021): ‘For me, doing at least 30 minutes of MVPA a day on most days of the week in my free time or when travelling is something…’ Participants answered on scales from 1 (*unpleasant, boring*) to 7 (*pleasant, fun*). Items were averaged (*r* = .55).

#### Self‐efficacy measures

Self‐efficacy was assessed at two levels in relation to its conceptual definition—i.e., the perceived capability to perform the target behaviour (Bandura, 1977)—using the operationalization ‘able to’ (see Williams & Rhodes, 2023, for a review). Both measures used single items (Cheval et al., 2017) with responses on a 7‐point scale from 1 (*totally incapable*) to 7 (*totally capable*). Self‐efficacy towards the exercise session was measured five minutes after the session with: ‘How capable did you feel of doing the exercises during the session?’ Self‐efficacy towards PA was measured 24–48 h after the exercise session with: ‘To what extent do you feel able to do at least 30 min of MVPA per day on most days of the week in your free time or while travelling?’

#### Physical activity outcomes

Daily PA was measured using a three‐axis accelerometer (Movisens Move4), worn on the hip, for eight consecutive days (yeilding seven full days of recording following the session). Participants were instructed to wear the device during waking hours and to remove it overnight. Daily data were considered valid if the device was worn for at least 10 waking hours per day (Evenson & Terry, 2009) and for at least 4 days, including one weekend day (Matthews et al., 2012). Wear time was computed using Data Analyzer software (v.1.13.5; movisens GmbH, Karlsruhe, Germany). PA intensities were classified according to their respective metabolic equivalent (MET) values (Ainsworth et al., 2000; Tremblay et al., 2017). Mean daily time spent in light (1.5–2.9 METs), moderate (3–6 METs) and vigorous (> 6 METs) intensity PA was used as an indicator of PA levels. Mean daily time spent in MVPA served as the primary dependent variable in the primary analyses. Mean daily total PA (i.e., light PA and MVPA) served as the dependent variable in the secondary analyses.

#### Potential confounders

A confounder is a variable associated with both the independent variable and the dependent variable, whose presence may falsely accentuate the relationship between them (Meinert, 1986). Potential confounders were therefore selected as variables theoretically associated with both ARs and MVPA. Based on prior work, the following potential confounders were identified: age, gender, BMI, past PA and perceived exertion (Bianchin & Angrilli, 2012; Cheval & Boisgontier, 2021; Ekkekakis et al., 2016; Lee et al., 2021; Maltagliati et al., 2022; Stojanovic et al., 2024). Age, gender, height and weight were self‐reported due to the limited availability of equipment for direct measurement in the community‐based exercise and health structures. BMI was calculated by dividing self‐reported weight (kg) by height^2^ (m). Past PA level was assessed using the Global Physical Activity Questionnaire (GPAQ; Armstrong & Bull, 2006). This questionnaire measures the frequency, duration and intensity of PA across different domains of daily life (work, transport and leisure), with time spent in MVPA as the primary indicator. Perceived exertion during the exercise session was measured with a single item: ‘How much effort are you exerting right now?’ (RPE, Foster et al., 2001). Participants answered on a scale from 0 (*no effort at all*) to 10 (*maximal effort*). The RPE was measured four times during the exercise session, directly after the ARs. RPE is widely accepted as a valid and easy‐to‐administer alternative for estimating exercise intensity in applied contexts, without requiring exercise testing prior to the exercise session (Bok et al., 2022). The intensity of the exercise sessions was modelled based on RPE, with RPE < 4 corresponding to light intensity, RPE 4–5 to moderate intensity and RPE > 5 to high intensity (Bishop et al., 2025).

A control variable assessing non‐specific treatment effects of the coach across exercise sessions (Hutchinson et al., 2023) was measured using the following four items: ‘I trust the coach to suggest appropriate exercises’, ‘The coach was very friendly’ ‘The coach listened carefully to the needs and difficulties I expressed during the session’, and ‘I feel that what the coach did and said in this session helped me to make progress’. Participants answered on a scale from 1 (*strongly disagree*) to 7 (*strongly agree*). Twenty‐four to forty‐eight hours after the exercise session, participants were invited to complete an online questionnaire and two reaction time tasks.^1^ These included self‐reported past PA levels and instrumental attitudes towards PA. A detailed description of these measures is provided in Data S3.

### Statistical analyses

All statistical analyses were performed using R (v.4.2.3). Patterns of missingness were assessed using Little's missing completely at random (MCAR) test (Little, 1988) and visual inspection. As no specific patterns were identified, missing data were handled using multiple imputation via the Multivariate Imputation by Chained Equations (MICE) R package (van Buuren & Groothuis‐Oudshoorn, 2011). The variables to be used in the analyses (i.e., ARs, remembered and forecasted pleasure, affective and instrumental attitudes, perceived exertion, self‐efficacy, intentions, age, gender, BMI, past PA and accelerometer‐based PA) were standardized first before being added to the dataset for imputation (Blomberg & Todorov, 2025). Descriptive statistics were conducted on non‐imputed data only. Ten imputed datasets were generated using default methods appropriate for continuous, binomial and multinomial variables (van Buuren & Groothuis‐Oudshoorn, 2011). The number of datasets to be imputed (*m* = 10) was determined based on the average percentage of missingness across all variables included in the imputation model (11%; Blomberg & Todorov, 2025). Pooling rules were then applied to combine the results from the imputed datasets for the final inference.

Model assumptions, including normality of residuals, linearity, multicollinearity and undue influence, were assessed using the Performance R package (Lüdecke et al., 2021). All assumptions were met. Both univariable and multivariable outliers were also detected and processed using this package.

#### Primary analyses

After conducting descriptive analyses, univariable linear regressions were performed to examine the association between the average AR (*H*_1_), the four AR scores (*H*_2_) and subsequent daily MVPA. Given that 53% of the sample had multimorbidity (i.e., ≥ 2 chronic diseases), with considerable overlap in the types of conditions within participants, conducting exploratory subgroup analyses based on chronic condition was considered impractical. For example, five participants reported osteoarthritis, a condition associated with pain and reduced mobility (Hunt & Papathomas, 2020), alongside less symptomatic conditions such as hypercholesterolaemia (Truong et al., 2016) (Figure S6). Given the complex patterns of multimorbidity and the limited sample size within each potential subgroup, we aggregated data across all chronic diseases to maximize statistical power and support more robust analyses. In addition, this approach allows us to test the extent to which affective mechanisms of PA engagement generalize across the heterogeneous and condition‐specific experiential constraints that characterize chronic diseases.

To determine which AR scores had the strongest association with MVPA (*H*_2_), we compared the overlap of the 95% confidence intervals (CI) of the standardized beta coefficients (*β*) using the ‘inferences by eyes’ method (Cumming & Finch, 2005). When the vertical distance between the upper CI of the lowest mean and the lower CI of the highest mean overlapped by less than 50%, *β* could be considered significantly different from each other, with *p* < .05. The method is described in detail in Data S4.

#### Exploratory analyses

Due to our limited sample size, potential confounders were filtered by examining a correlation matrix, with only variables correlated with both MVPA and ARs included in the final multivariable models. Only BMI and RPE_3_ met these criteria (Data S5, Table S2). Multivariable linear regression models were then performed to control for the identified confounders (*EA*_*1*_).

Exploratory serial mediation models were computed to test the reflective affect processing pathway (AR → remembered pleasure → forecasted pleasure → affective attitudes → subsequent daily MVPA) (*EA*_2_)^2^ and the self‐efficacy pathway (AR → self‐efficacy towards exercise → self‐efficacy towards PA → subsequent daily MVPA) (*EA*_3_) (Figure 1), while controlling for confounders (i.e., BMI and RPE_3_). The method is described in detail in Data S6.

#### Secondary analyses

Secondary analyses were conducted for all primary and exploratory hypotheses using mean daily total PA—encompassing light‐intensity PA and MVPA—as the dependent variable. Their purpose is twofold. First, they aim to provide a more comprehensive evaluation of potential spillover effects across the full spectrum of PA intensities, beyond the MVPA threshold targeted in the primary analyses. Second, they address the conceptual limitation that a substantial proportion of exercise sessions in the present sample were conducted at intensities below the MVPA threshold, which may have attenuated the association between in‐session ARs and subsequent MVPA. All secondary analyses follow the same analytical procedures as the corresponding primary and exploratory analyses, with total PA substituted for MVPA as the dependent variable.

## RESULTS

Results are presented in several parts. First, descriptive results summarize the characteristics of the sample and the exercise sessions. Next, analytical results are organized by hypothesis. For each hypothesis, primary analyses examining subsequent daily MVPA are presented first, followed by secondary analyses examining subsequent daily total PA, which should be considered exploratory.

### Descriptive results

Table 1 shows the demographic and health characteristics of the participants. Most of them were women (79.41%), older adults (M_age_ = 66.56 ± 12.55 years), and classified as overweight (M_BMI_ = 26.46 ± 5.84 kg/m^2^). A significant proportion (88.1%) reported having at least one chronic disease. Participants reported high levels of intention to engage in daily PA (M_intention_ = 5.53 ± 1.65 out of 7) and self‐reported high levels of past MVPA (M_MVPA_ = 9.94 ± 20.77 h/week). Accelerometer‐based measures of MVPA showed that participants spent an average of 4.59 h per week in MVPA, equivalent to 39.38 (± 24.48) min per day during the week following the exercise session (Table 1). Based on accelerometer data, 72.50% of participants met the World Health Organization's (WHO) recommendation of 150 min of weekly MVPA. Table 2 shows the variables that were measured during and immediately after the exercise session. ARs, remembered pleasure and forecasted pleasure were predominantly positive (FS_mean_ = 3.00 ± 1.38; M_remebered_ = 3.47 ± 1.50; M_forecasted_ = 3.41 ± 1.52), and perceived exertion was generally low (RPE_mean_ = 2.45 ± 1.33) (Table 2, Figure S10). Coaches were rated highly in terms of trustworthiness, friendliness, support for participants' needs and facilitating progress (M_trust_ = 6.9 ± 0.4; M_friendly_ = 6.9 ± 0.5; M_needs_ = 6.8 ± 0.6; M_progress_ = 6.5 ± 0.9; out of 7).

**TABLE 2:** Mean exercise‐related variable during and after the exercise session.

Variables	Mean (SD)	Missing (%)
Affective responses [−5–5]		
FS_1_	3.34 (1.63)	7 (6.42)
FS_2_	3.02 (1.69)	7 (6.42)
FS_3_	2.57 (2.08)	7 (6.42)
FS_4_	3.08 (1.90)	7 (6.42)
FS_mean_	3.00 (1.38)	7 (6.42)
Perceived exertion [0–10]		
RPE_1_	1.80 (1.56)	7 (6.42)
RPE_2_	2.75 (1.52)	7 (6.42)
RPE_3_	3.58 (1.95)	7 (6.42)
RPE_4_	1.66 (2.10)	8 (7.34)
RPE_mean_	2.45 (1.33)	7 (6.42)
Global perceived exertion [0–10]	3.38 (1.50)	17 (15.60)
Remembered pleasure [−5–5]	3.47 (1.50)	17 (15.60)
Forecasted pleasure [−5–5]	3.41 (1.52)	17 (15.60)
Self‐efficacy towards exercise [1–7]	5.76 (1.30)	17 (15.60)
Intention towards PA [1–7]	5.53 (1.63)	17 (15.60)
Coach rating [1–7]		
Trust	6.9 (0.4)	17 (15.60)
Friendly	6.9 (0.5)	17 (15.60)
Needs	6.8 (0.6)	17 (15.60)
Progress	6.5 (0.9)	17 (15.60)

Abbreviations: FP, forecasted pleasure; FS, Feeling Scale; MVPA, moderate‐to‐vigorous physical activity; PA, physical activity; RP, remembered pleasure; RPE, rating of perceived exertion.

### Association between affective responses and subsequent daily physical activity ( *H*_1_ _–2_)

#### Moderate‐to‐vigorous physical activity

**FIGURE 3:**
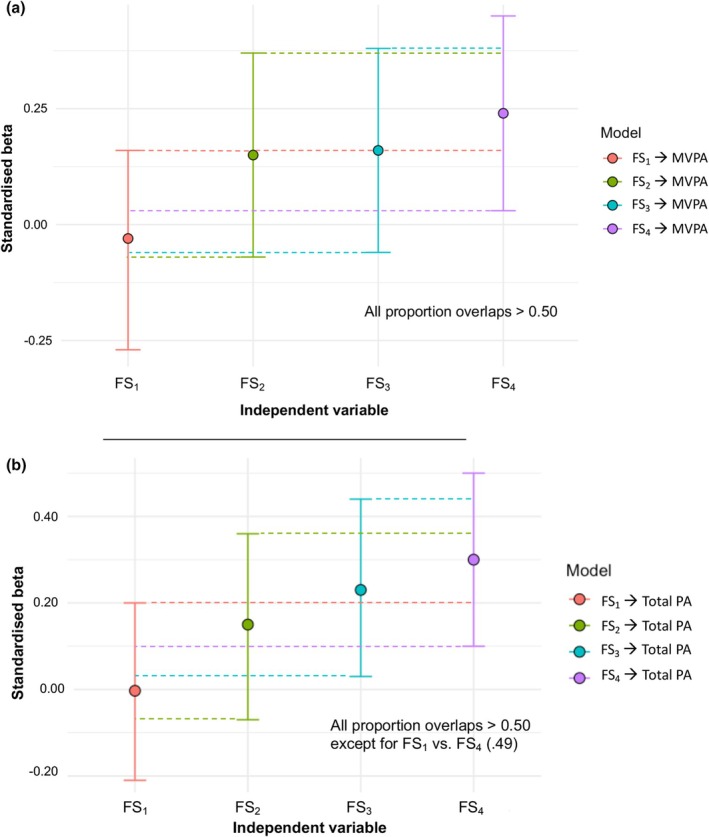
Difference in effect sizes across the associations between affective response at different times and subsequent daily time spent in MVPA and total PA. FS, Feeling Scale; MVPA, moderate‐to‐vigorous physical activity; Total PA, total physical activity (including light PA and MVPA). Panel A = MVPA, Panel B = Total PA. FS was measured four times during an exercise session: at the start of the warm‐up (FS_1_), at the start of the first exercise (FS_2_), at the start of the last exercise (FS_3_), and and at the last minute of the session (FS_4_). If the proportion of overlap of the 95% confidence intervals (CIs) is > 0.50, this suggests that the difference in the coefficients is not significant (*p* > .05).

Contrary to the spillover effect hypothesis (*H*_1_), univariable linear regression analysis showed no significant association between the average AR during the exercise session and subsequent daily MVPA (*b* = 4.76, 95% CI [−0.29; 9.81], *R* ^2^ = .04, *p =* .064). In line with our second hypothesis (*H*_2_), results showed a significant positive association between the last AR (FS_4_) and subsequent daily MVPA (*b* = 5.84, 95% CI [0.26; 11.42], *R* ^2^ = .06, *p =* .041). Conversely, no significant associations were observed for ARs measured at earlier moments during the session (Table S2). Although FS_4_ showed a larger effect size than FS_1_, FS_2_ and FS_3_, comparisons of the CIs using the ‘inferences by eye’ method showed no significant differences (*p* > .05; Figures 3 and S7).

#### Total physical activity

Secondary analyses showed a significant association between the average AR (*b* = 15.41, 95% CI [2.61; 28.21], *R* ^2^ = .06, *p =* .019), the AR measured at the start of the last exercise (FS_3_; *b* = 14.62, 95% CI [0.52; 28.72], *R* ^2^ = .06 *p =* .042), the last AR (FS_4_; *b* = 18.07, 95% CI [4.84; 31.31], *R* ^2^ = .09 *p =* .008), and subsequent daily total PA. Neither FS_1_ nor FS_2_ was significantly associated with subsequent daily total PA. While FS_3_ and FS_4_ showed larger effect sizes than FS_1_ and FS_2_, only FS_4_ differed significantly from FS_1_ (*p* < .05; Figure 3).

### Testing for spillover effect after adjustment for potential confounders ( *EA*_1_ )

#### Moderate‐to‐vigorous physical activity

After adjusting for BMI and RPE_3_ in the multivariable regression analysis (*EA*_1_), FS_4_ was no longer significantly associated with subsequent daily MVPA (*p* = .372; Table 3). The results revealed that BMI was significantly associated with subsequent daily MVPA (*b* = −7.23, 95% CI [−12.85; −1.61], *p* = .013), but not RPE_3_ (*p* = .251; Table 4).

**TABLE 3:** Association between affective response at different times and subsequent daily time spent in MVPA and total PA.

	b (95% CI)	*ß* (95% CI)	*R* ^2^	*p*
Model 1 (MVPA)				
Intercept	40.53 (35.67; 45.39)	.00 (−.22; .22)	.04	< .001
FS_mean_	4.76 (−0.29; 9.81)	.18 (−.03; .39)		.064
Intercept	40.18 (34.58; 45.79)	.01 (−.22; .22)	.002	< .001
FS_1_	−0.46 (−6.41; 5.47)	−.05 (−.27; .16)		.874
Intercept	40.24 (34.76; 45.72)	.01 (−.20; .22)	.03	< .001
FS_2_	4.12 (−0.99; 9.28)	.15 (−.07; .37)		.113
Intercept	40.26 (34.72; 45.81)	.01 (−.20; .22)	.02	< .001
FS_3_	3.85 (−1.23; 8.94)	.16 (−.06; .38)		.136
Intercept	40.20 (34.80; 45.59)	.00 (−.21; .21)	.06	< .001
FS_4_	5.84 (0.26; 11.42)	.24 (.03; .45)		.041
Model 2 (total PA)				
Intercept	142.86 (131.04; 154.70)	.03 (−.21; .27)	.06	< .001
FS_mean_	15.41 (2.61; 28.21)	.28 (.04; .53)		.019
Intercept	144.00 (126.88; 161.12)	−.004 (−.22; .21)	.001	< .001
FS_1_	0.67 (−12.39; 13.73)	−.003 (−.21; .20)		.919
Intercept	143.98 (126.76; 161.19)	−.003 (−.21; .22)	.02	< .001
FS_2_	9.43 (−2.77; 21.62)	.15 (−.07; .36)		.128
Intercept	144.05 (127.01; 161.08)	−.002 (−.21; .20)	.06	< .001
FS_3_	14.62 (0.52; 28.72)	.23 (.03; .44)		.042
Intercept	143.98 (127.49; 160.47)	−.005 (−.21; .20)	.09	< .001
FS_4_	18.07 (4.84; 31.31)	.30 (.10; .50)		.008

*Note*: FS was measured four times during an exercise session: at the start of the warm‐up (FS_1_), at the start of the first exercise (FS_2_), at the start of the last exercise (FS_3_) and at the last minute of the session (FS_4_). FS_mean_ represents the mean score of the four FS scores. *N* = 109.

Abbreviations: S, Feeling Scale; MVPA, moderate‐to‐vigorous physical activity; Total PA, all physical activity intensities.

**TABLE 4:** Association between affective response at different times and subsequent daily time spent in MVPA and total PA controlling for confounders.

	b (95% CI)	*ß* (95% CI)	*R* ^2^ adj.	*p*
Model 1 (MVPA)				
Intercept	40.12 (34.87; 45.36)	.00 (−.10; .20)		<.001
FS_4_	2.61 (−3.23; 8.44)	.11 (−.10; .33)		.372
BMI (kg/m^2^)	−7.23 (−12.85; −1.61)	−.30 (−.50; −.09)		.013
RPE_3_	−3.01 (−8.21; 2.18)	−.12 (−.33; .09)		.251
Total *R* ^2^ adj.			.16	
Model 2 (Total PA)				
Intercept	143.00 (131.19; 154.80)	.03 (−.21; .27)		<.001
FS_mean_	12.93 (−0.51; 26.38)	.23 (−.03; .49)		.059
BMI (kg/m^2^)	−10.42 (23.83; 2.99)	−.18 (−.42; .07)		.126
RPE_3_	4.51 (−8.40; 17.43)	−.03 (−.18; .25)		.489
Total *R* ^2^ adj.			.09	
Intercept	143.79 (126.72; 160.87)	.01 (−.21; .20)		<.001
FS_3_	12.31 (−2.77; 27.38)	.19 (−.03; .41)		.107
BMI (kg/m^2^)	−13.68 (−27.08; −0.27)	−.22 (−.43; .001)		.046
RPE_3_	6.14 (−7.36; 19.64)	−.09 (−.12; .31)		.367
Total *R* ^2^ adj.			.10	
Intercept	143.73 (127.15; 160.30)	.01 (−.21; .19)		<.001
FS_4_	16.76 (3.10; 30.43)	.28 (.05; .50)		.017
BMI (kg/m^2^)	−12.84 (−26.14; 0.46)	−.20 (−.42; .02)		.058
RPE_3_	7.83 (−5.32; 20.97)	.12 (−.09; .33)		.239
Total *R* ^2^ adj.			.13	

*Note*: FS and RPE were measured four times during an exercise session: at the start of the warm‐up (FS_1_, RPE_1_), at the start of the first exercise (FS_2_, RPE_2_), at the start of the last exercise (FS_3_, RPE_3_) and at the last minute of the session (FS_4_, RPE_4_). *N* = 109. Only the independent variables that were significantly associated with MVPA and total PA in the univariate analyses are presented.

Abbreviations: BMI, body mass index; FS, Feeling Scale; MVPA, moderate‐to‐vigorous physical activity; Total PA, all physical activity intensities; RPE, rating of perceived exertion.

#### Total physical activity

Secondary analyses showed that after adjusting for BMI and RPE_3_, only FS_4_ remained significantly associated with subsequent daily total PA, whereas associations with average AR and FS_3_ were no longer significant (*p*s > .05; Table 4).

### Underlying mechanisms of the association between affective responses and subsequent daily physical activity

The following results focus on the last AR, as this was the only individual AR significantly associated with daily MVPA in the univariable analysis (*H*_2_). Although the association between the last AR and daily MVPA became non‐significant after adjusting for confounders, we proceeded with exploratory serial mediation analyses, including these confounders, to provide a comprehensive assessment (*EA*_2_ and *EA*_3_). All models were also tested using the other ARs and the average AR and are presented in Data S6.

#### Reflective affect processing pathway ( *EA*_2_ )

##### Moderate‐to‐vigorous physical activity

**FIGURE 4:**
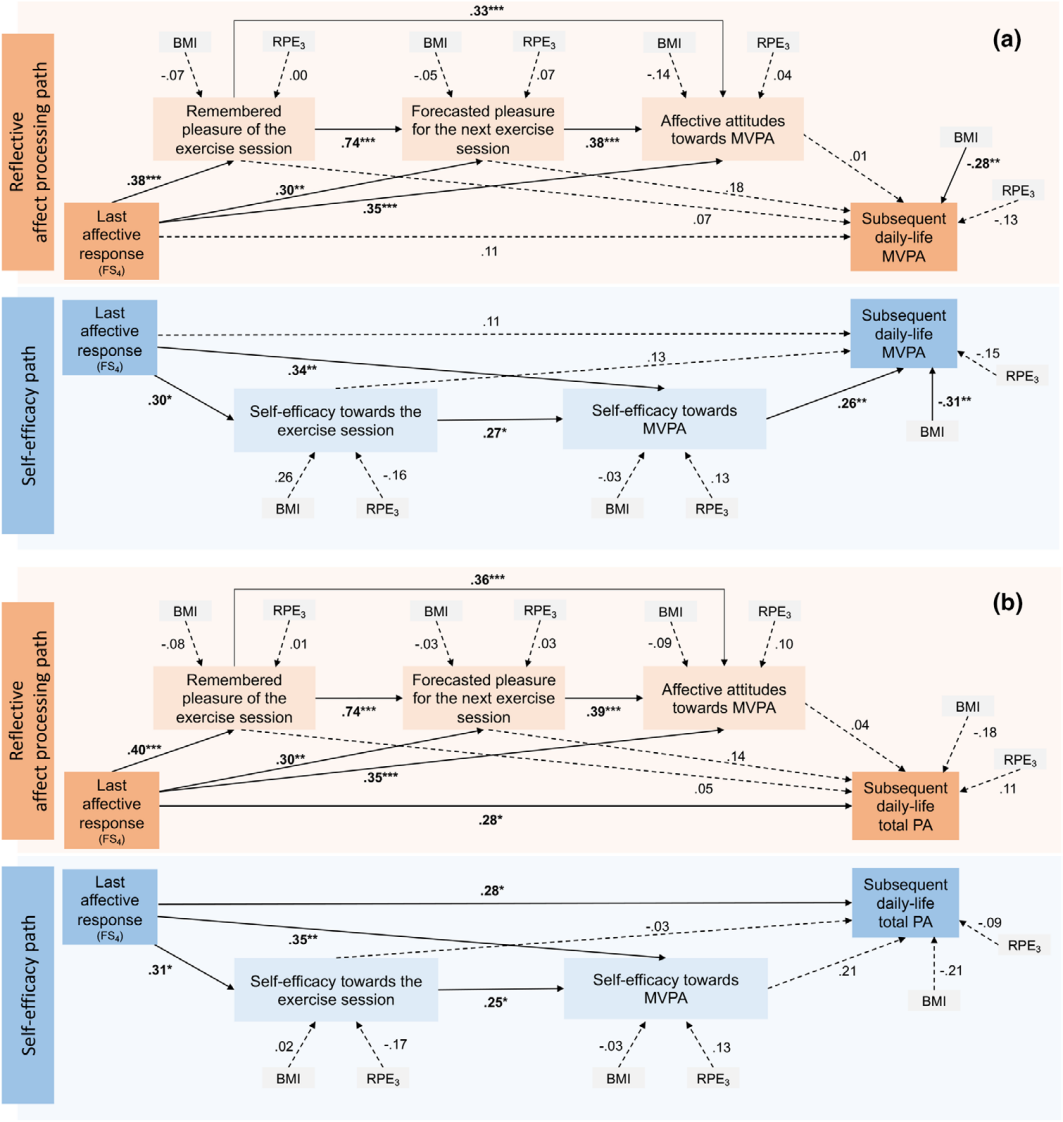
Serial mediation analyses for MVPA and total PA. BMI, body mass index; MVPA, moderate‐to‐vigorous physical activity; PA, total physical activity (including light PA and MVPA); RPE_3_, perceived exertion measured at the start of the last exercise of the session. Panel A = MVPA, Panel B = Total PA. *p < .05; **p < .01; ***p < .001. *N* = 109. The coefficients represent standardized betas.

Exploratory serial mediation analyses showed that (a) the last AR significantly predicted remembered pleasure (*ß* = .38, *p* < .001), which in turn (b) significantly predicted forecasted pleasure (*ß* = .73, *p* < .001), which (c) significantly predicted affective attitudes towards daily MVPA (*ß* = .31, *p* = .028). The final step of this pathway, however, was not supported: affective attitude was not significantly associated with daily MVPA (*ß* = .01, *p* = .912), when controlling for BMI and RPE_3_ (Figure 4). Consequently, no significant indirect effect of the last AR on daily MVPA via the reflective affect processing pathway was found (see Data S6, Table S3). Similar patterns were observed for the average AR and previous ARs, with no significant differences in effect size on the association between ARs and remembered pleasure (*p* > .05; Data S4, Figure S8).

##### Total physical activity

Secondary analysis showed a similar serial mediation pattern with total PA, with one notable difference: unlike MVPA, the direct effect of the last AR on daily total PA was significant (*ß* = .28, *p* = .017, Figure 4).

#### *Self‐efficacy pathway* ( *EA*_3_ )

##### Moderate‐to‐vigorous physical activity

Exploratory serial mediation analyses showed that (a) the last AR significantly predicted self‐efficacy towards the exercise session (*ß* = .30, *p* = .007), which in turn (b) significantly predicted self‐efficacy towards MVPA (*ß* = .27, *p* = .014), which (c) significantly predicted daily MVPA (*ß* = .26, *p* = .014), when controlling for BMI and RPE_3_ (Figure 4). Results showed a small but statistically significant indirect effect of the last AR on daily MVPA via self‐efficacy towards MVPA (*ß* = .05, 95% CI [.002; .12]; Data S6, Table S4).

##### Total physical activity

Secondary analysis showed a broadly similar serial mediation pattern with total PA. However, two differences were observed relative to the primary analyses. First, self‐efficacy towards MVPA did not significantly predict subsequent daily total PA. Second, the direct effect of the last AR on daily total PA is now significant (*ß* = .28, *p* = .017, Figure 4).

## DISCUSSION

This study aimed to examine whether ARs assessed at specific points during an exercise session could predict subsequent daily MVPA in PCD, and to explore two potential pathways—reflective affect processing and self‐efficacy—through which ARs may predict PA behaviour. Secondary analyses additionally examined total PA as a complementary outcome. Understanding these relationships is critical given the high prevalence of physical inactivity in this population and the need for theory‐driven interventions to promote sustained PA engagement.

### Main findings

Only the last AR predicted subsequent daily MVPA, suggesting that higher AR at the end of an exercise session is associated with higher MVPA during the following week in PCD. However, effect sizes did not significantly differ across the various ARs. Critically, the association between the last AR and daily MVPA became non‐significant after adjusting for BMI and RPE_3_ (*EA*_1_). In contrast, secondary analyses showed significant positive associations between the last AR and subsequent daily total PA, even after controlling for confounders, suggesting that the spillover effect may operate more consistently across the full spectrum of PA intensities. Exploratory mediation analyses provided partial support for the reflective affective processing pathway (AR → remembered pleasure → forecasted pleasure → affective attitudes). However, affective attitudes did not predict MVPA after adjusting for covariates (*EA*_2_). By contrast, the self‐efficacy pathway received stronger support for MVPA (*EA*_3_): the last AR predicted exercise self‐efficacy, which in turn predicted higher self‐efficacy towards MVPA, which predicted higher levels of MVPA even after controlling for confounders. While statistically significant, the indirect effect was small in standardized terms (*β* = .05). This corresponds to an estimated increase of approximately 1.22 min of daily MVPA (95% CI [0.05, 2.94]) for every one‐standard‐deviation increase in the last AR, operating through self‐efficacy towards MVPA. Secondary analyses on total PA revealed both similarities and differences relative to the primary analyses, which are discussed in detail below.

### Comparison with other studies

Univariable regressions showed that only the last AR of the exercise session significantly and positively predicted subsequent daily MVPA. FS_4_ was assessed during the final minute of the session—immediately preceding the coach's formal termination—during an active exercise phase, which precludes the possibility of a recovery‐related affective rebound. Furthermore, consistent with the peak–end rule, the affective experience at the end of an event influences the global evaluation of that experience regardless of the intensity at that moment. The decline in perceived exertion from RPE_3_ to RPE_4_ likely reflects a natural reduction in exercise intensity towards session termination. Whether this reduction constitutes an active cool‐down depends on the definition adopted, as no consensus exists on this point—some authors define it as a separate activity following the session (Van Hooren & Peake, 2018), while others consider it an integral part of the session itself (Franklin et al., 2022).

Neither the average AR nor the ARs assessed at other moments during the exercise session (i.e., at the start of the warm‐up and at the start of the first and last exercises) were significantly associated with MVPA (*H*_1_). Although traditional binary statistical decisions may imply conflicting results (Amrhein et al., 2019), the correlation observed for the average AR (*r =* .18, *p =* .064) falls within the lower range of effect sizes reported in previous studies (*r =* .24 to *r =* .51; Williams et al., 2008; Williams et al., 2012), suggesting broad consistency despite not reaching statistical significance. Although the last AR showed the strongest predictive value for MVPA, the differences in effect sizes did not reach statistical significance. Similarly, no significant differences were observed in the association between ARs and remembered pleasure across time points, providing limited support for the peak–end rule in this ecological context. Although Kahneman (2000) showed that remembered utility shaped by end affect can guide behavioural choices, our results suggest that in habitual exercisers engaging in real‐world exercise sessions, end affect does not appear to carry disproportionate weight in the remembered pleasure compared to other moments. Taken together, these findings provide only limited support for both the hedonic principle (Cabanac, 1992; Williams, 2008) and the peak–end rule in predicting MVPA among PCD. However, these results should be interpreted with caution, as MVPA accounted for a relatively small proportion of total PA in our sample, which may have contributed to the attenuation of these associations.

Multivariable analyses showed that the association between the last AR and subsequent daily MVPA was no longer significant after adjusting for BMI and perceived exertion, consistent with findings in healthy samples (Williams et al., 2008, 2012). These results suggest that previously reported spillover effects from exercise‐induced ARs to daily MVPA may partly reflect unaccounted confounding rather than a direct behavioural influence of a single exercise session. However, these results should be interpreted cautiously due to the limitations of accelerometers in distinguishing between exercise‐specific and general PA, and the exploratory nature of *EA*_1_. In addition, exercise intensity was modelled using perceived exertion rather than physiological measures. According to Dual‐Mode Theory (Ekkekakis, 2003), exercise intensity plays a central role in shaping ARs. At light intensity (below the first metabolic threshold, RPE < 4, Bishop et al., 2025), ARs tend to be homogeneously positive. At moderate intensity (between the two metabolic thresholds, RPE 4–5), ARs exhibit substantial inter‐individual variability. At high intensity (above the second metabolic threshold, RPE > 5), ARs are homogeneously negative. In the present study, perceived exertion was relatively low, and ARs were predominantly positive, suggesting that most exercise sessions occurred within an intensity associated with limited affective variability (Table S1, Figure S5). Notably, perceived exertion showed greater variability at the third measurement point (RPE_3_), with some sessions reaching moderate intensity—an intensity associated with substantial inter‐individual variability in ARs according to Dual‐Mode Theory (Figure S10). This may partly explain why RPE_3_ emerged as a significant confounder in the multivariable analyses. However, the absence of laboratory‐based physiological markers and continuous monitoring restricts our ability to model exercise intensity and determine whether ARs primarily reflect hedonic appraisal or differences in physiological load. Future studies should therefore employ physiological markers (e.g., metabolic threshold for aerobic exercise) to model exercise intensity. Such rigorous measurement would enable researchers to directly test the hypothesis that exercise intensity moderates the AR–PA relationship, particularly by comparing affective spillover across light, moderate and high‐intensity exercise sessions. Furthermore, it is worth noting that Dual‐Mode Theory and the peak–end rule were largely developed and tested in continuous exercise contexts. Whether their predictions generalize to interval‐based exercise, which represented 85% of sessions in the present study, remains an open question for future research.

Individual‐level variables, such as BMI, are likely to predict both ARs during exercise sessions and daily MVPA (Biddle et al., 2021; Ekkekakis et al., 2016), creating spurious associations. For example, people with higher BMI may experience greater physical discomfort during exercise, which may negatively predict their ARs (Ekkekakis et al., 2010, 2016). At the same time, higher BMI often creates physical barriers to activity (Biddle et al., 2021; Ekkekakis et al., 2016), resulting in lower daily MVPA. Thus, BMI may act as a common cause of both lower ARs and lower MVPA, producing an observed association between these variables that disappears once BMI is statistically controlled. While this confounding mechanism is likely to operate across populations, it may be particularly pronounced in PCD, where factors such as chronic pain, discomfort, or exercise‐related anxiety (e.g., Aydemir et al., 2022; Goubran et al., 2025) can further constrain both affective experiences during exercise and daily PA capacity. However, the critical methodological insight from our study—corroborated by findings in healthy samples (Williams et al., 2008, 2012)—is that apparent spillover effects may disappear when appropriate confounders are controlled, regardless of population characteristics.

Another possible explanation for the null findings after confounder adjustment lies in the cumulative ‘dose’ of ARs across multiple exercise sessions, as the ARs from a single session may be insufficient to significantly impact behaviour (Stevens et al., 2020). For example, a recent study on cardiovascular patients showed that the accumulation of positive affective exercise experiences related to exercise was positively associated with attraction towards PA, which in turn positively predicts self‐reported PA levels (Piva et al., 2025). Given the long‐term exercise history of our sample (average of 6 years), it is possible that the accumulation of exercise‐related affective experiences has already shaped more established determinants of PA behaviour—such as self‐efficacy and exercise habit—which are more strongly associated with daily MVPA than session‐specific exercise‐induced ARs (Bandura, 1977; Rhodes & Sui, 2021; Weyland et al., 2022). This is further supported by the significant association between self‐efficacy towards MVPA and subsequent daily MVPA, suggesting that daily MVPA in our sample is driven primarily by these higher‐order processes rather than by the ARs of a single session. Future studies should examine this spillover effect in more diverse and less active populations and explicitly test dose–response relationships between repeated affective exercise experiences and daily MVPA.

Although the direct association between ARs and daily MVPA was not supported after confounder adjustment, our exploratory analyses examined whether ARs might predict MVPA indirectly through cognitive and affective mediators. To the best of our knowledge, this study is the first formal comparison of the reflective affect processing pathway, as modelled in the AHBF, and the self‐efficacy pathway, grounded in Bandura's self‐efficacy theory (Bandura, 1977), as called for by Stevens et al. (2020). Although exploratory, the reflective affect processing pathway was not fully supported by our analyses. Notably, our study supports and extends previous research by highlighting the association between ARs and remembered pleasure in a population with chronic disease in an ecological setting (Bastos et al., 2025; Hutchinson et al., 2023; Zenko et al., 2024). Furthermore, results showed that remembered pleasure predicted forecasted pleasure, which in turn predicted affective attitudes—a relationship that has been theorized (Fredrickson, 2000; Jones & Zenko, 2021) but rarely demonstrated empirically. Despite the well‐documented association between affective attitudes and MVPA (Ajzen, 1991; Bermudez et al., 2021; Rhodes et al., 2019), our results did not identify such a relationship. The near‐zero effect sizes observed (*β* = .01 for MVPA and *β* = .04 for total PA) suggest that this absence is unlikely to reflect insufficient statistical power, but is instead consistent with the pattern described above: in long‐term exercisers, MVPA may be primarily governed by more established determinants such as self‐efficacy and exercise habit rather than by affective attitudes towards PA. The role of exercise habit in this context remains speculative, and future research should examine whether habit moderates or mediates the relationship between session‐specific affective responses and subsequent PA in long‐term exercisers.

Exploratory serial mediation analyses provided preliminary support for the self‐efficacy pathway, suggesting that the last AR may predict PA behaviour through self‐efficacy as a cognitive mediator. Consistent with Bandura's theory (1997), which identifies affective and physiological states as a foundational source of self‐efficacy beliefs, our results suggested that experiencing positive affective states during exercise may enhance self‐efficacy towards both exercise and MVPA, which in turn predicted higher levels of daily MVPA. Although exploratory, this finding aligns with a substantial body of research in exercise psychology demonstrating the critical role of self‐efficacy in PA engagement and maintenance (McAuley, 1993; McAuley & Blissmer, 2000). However, given the small magnitude of the indirect effect and the exploratory nature of these analyses, these findings should be considered preliminary and interpreted with caution. Moreover, previous studies have shown bidirectional relationships between ARs and self‐efficacy during exercise, with self‐efficacy moderating ARs to acute exercise (Focht et al., 2007; McAuley et al., 1995) and ARs influencing subsequent self‐efficacy beliefs (McAuley et al., 1999). Although Rhodes and Kates (2015) found mixed evidence for the relationship between ARs and self‐efficacy in their systematic review, our findings suggest that this pathway may be particularly relevant in PCD, where self‐efficacy has been identified as a critical determinant of PA behaviour (Johansson et al., 2022; Selzler et al., 2020). The self‐efficacy pathway may be especially important in this population, as chronic disease symptoms and functional limitations can create substantial barriers to PA engagement that must be overcome through enhanced confidence in one's capabilities. However, given the small magnitude of the indirect effect and the exploratory nature of these analyses, these findings should be considered preliminary and interpreted with caution.

To the best of our knowledge, this is the first study to examine associations between exercise‐induced ARs and subsequent daily total PA in PCD. These secondary analyses align with recent recommendations to include light‐intensity PA when assessing overall activity levels, particularly in PCD (Lee et al., 2025). Unlike the primary analyses, secondary analyses showed that total PA was positively associated with the last AR even after adjusting for confounders. However, no evidence was found to support the self‐efficacy pathway for the last AR in these secondary analyses. At least two factors may explain these divergences between primary and secondary analyses. First, a substantial proportion of participants engaged in exercise sessions at intensities below the MVPA threshold. This structural misalignment between the intensity of the exercise sessions and the primary outcome—MVPA—may have attenuated the association between ARs and MVPA, whereas total PA captures a broader spectrum of activity levels more consistent with the intensity profile of the exercise sessions. Second, the non‐significant association between self‐efficacy towards MVPA and total PA likely reflects a correspondence problem between the specificity of the self‐efficacy measure (towards MVPA) and the behavioural outcome (total PA) (Ajzen & Timko, 1986; Irving & Smith, 2020).

### Limitations and strengths

The current study has several limitations. First, and most centrally, the final sample of 109 participants resulted in 73% power to detect the smallest effect size reported in the literature (*f* ^2^ = .06), falling below the conventional 80% threshold recommended by Cohen (1988). Although the study was adequately powered to detect effect sizes of *f* ^2^ = .10 or above—a range encompassing the effects reported in prior work—null findings, particularly those where the effect size falls below *f* ^2^ = .10, should be interpreted with caution, as they may reflect insufficient power to detect small effects. Conversely, significant findings that emerge from secondary analyses should be considered preliminary, given their exploratory nature. Second, accelerometer‐based MVPA does not differentiate between the various contexts in which PA behaviours occur. In other words, our primary dependent variable cannot tell whether PA was performed during a subsequent exercise session (i.e., in the same context) or during daily life (i.e., reflecting a true spillover effect). Although secondary analyses included all‐intensity PA to capture a broader range, these are not exempt from such limitations, as exercise behaviours can also occur at a light intensity. Third, the observational design of the study limits causal inference. Fourth, the present study only focused on the ‘end effect’ of the peak–end rule, overlooking the peak itself. This is mainly because we examined habitual exercise sessions within different exercise and health programmes, which made it difficult to control exercise intensity and, therefore, the peak across sessions. Consequently, each participant may experience their peak at a different time point. To detect a peak in such settings, future studies could rely on retrospective assessments of peak affect—in a manner analogous to global RPE—which, while potentially subject to recall bias, would avoid overburdening participants during exercise (Lackner et al., 2014). Alternatively, continuous physiological measures such as heart rate variability or cerebral oxygenation could provide a psychophysiological proxy for peak affect without requiring additional self‐report assessments (e.g., Jones & Ekkekakis, 2019; Karageorghis et al., 2025). Fifth, our sample consisted of PCD who were engaged in regular, supervised exercise over a long period, reporting high self‐efficacy, positive attitudes and strong intentions towards PA. Thus, our findings may not be generalizable to less motivated and less active PCD. Furthermore, ARs during exercise were predominantly positive, and perceived exertion was relatively low in our sample. This homogeneity suggests potential range restriction and ceiling effects, which may have reduced the variability in our predictors and limited the statistical sensitivity to detect small associations between ARs and subsequent MVPA. Future research should examine the potential spillover effect in inactive PCD populations, who may benefit more from increased PA, and for whom acute ARs may play a more critical role in PA initiation and maintenance (Anderson & Durstine, 2019; Rhodes & Sui, 2021). Finally, our sample included a heterogeneous range of chronic conditions, which complicates the interpretation of associations between ARs and subsequent PA. Symptom‐related constraints (e.g., pain, dyspnoea) may differentially shape interoceptive experiences during exercise, thereby influencing ARs, perceived exertion, self‐efficacy and subsequent PA, compared to largely asymptomatic conditions such as hypercholesterolaemia (Barhorst et al., 2020; Bernhardt et al., 2013; Thombs et al., 2017; Truong et al., 2016). For example, psychological and physiological limitations, such as fear of PA, claudication pain and reduced mobility, are particularly pronounced in patients with cardiovascular disease or arthritis (Abaraogu et al., 2018; Hoffmann et al., 2018; Hunt & Papathomas, 2020; Murphy et al., 2016), and may contribute to lower PA levels (Barker et al., 2019; Goubran et al., 2025). It is therefore possible that the heterogeneity of chronic conditions in our sample introduced additional variability in ARs, which may have attenuated the observed associations and partly contributed to the null or weak spillover effects observed in the present study. However, while symptom profiles vary, evidence suggests that the underlying psychological determinants of PA (e.g., self‐efficacy, intentions) and their associations with behaviour do not differ significantly across diverse chronic disease populations (Rhodes & Blanchard, 2007). These findings highlight the need for future research to further examine condition‐specific effects or the potential moderating role of symptom‐related constraints in the AR–PA relationship.

The study has several notable strengths. First, it extends existing literature by investigating the spillover effect of exercise‐induced ARs on daily MVPA and total PA and testing the ‘peak–end rule’ in PCD. Second, ARs were assessed in an ecological setting during participants' routine exercise sessions as part of their usual exercise and health programme. Third, daily MVPA and total PA were measured using accelerometers, which offer several advantages over self‐reported questionnaires: they provide direct, continuous measurement of PA behaviour without social desirability bias and have been shown to provide a more reliable estimate in small sample size studies (Grimm et al., 2012; Ogonowska‐Slodownik et al., 2022). Fourth, this study is the first to formally compare the reflective affect processing and self‐efficacy pathways. Finally, our models were adjusted for potential confounders, providing a more nuanced understanding of the relationship between exercise‐induced ARs and subsequent daily MVPA and total PA.

## CONCLUSION

In conclusion, this study suggests that the association between the last AR during a single exercise session and subsequent daily MVPA in PCD may be influenced by confounding factors, such as BMI, raising questions about the existence of a true spillover effect from session‐specific ARs to daily MVPA. Secondary analyses further showed that the last AR predicted total PA independently of confounders, a pattern consistent with the notion of an intensity‐specific spillover effect whereby positive affective experiences during an exercise session may increase the likelihood of engaging in daily‐life PA at similar intensity levels. However, given the exploratory nature of these analyses, this finding should be considered preliminary and interpreted with caution, particularly in light of the limitations of accelerometers in distinguishing between exercise‐specific and general PA. Additionally, the results indicate that ARs contribute to affective evaluations of MVPA and, more robustly, to self‐efficacy beliefs, which predict MVPA. The study provides limited support for the peak–end rule, as end affect did not show a stronger association with remembered pleasure than earlier ARs. Given the limited statistical power of the study and the exploratory nature of several analyses, caution is warranted in interpreting these conclusions. Future well‐powered studies should investigate how affective experiences in specific contexts, such as rehabilitation programmes, influence both session‐specific exercise behaviour and daily PA in PCD to inform the development of effective, theory‐driven PA interventions.

## AUTHOR CONTRIBUTIONS

**Layan Fessler:** Conceptualization; methodology; formal analysis; investigation; visualization; writing – original draft; writing – review and editing. **Dan Orsholits:** Formal analysis; writing – review and editing. **Alexis Le Faucheur:** Formal analysis; writing – review and editing. **Ryan E. Rhodes:** Conceptualization; writing – review and editing. **Boris Cheval:** Conceptualization; methodology; supervision; formal analysis; writing – original draft; writing – review and editing. **Philippe Sarrazin:** Conceptualization; methodology; formal analysis; supervision; writing – original draft; writing – review and editing.

## Supporting information


**Data S1.** Sample size calculation and sensitivity analyses.
**Figure S1.** A priori power analysis for *H*_1_ and *H*_2_.
**Figure S2.** Univariable regression analyses.
**Figure S3.** Association between affective responses and daily MVPA controlling for confounders.
**Figure S4.** Mediation analyses (path b and c').
**Data S2.** Exercise sessions and participants information.
**Table S1.** Exercise sessions' information.
**Figure S5.** Exercise sessions' characteristics.
**Figure S6.** Co‐occurrence between chronic conditions in participants with multimorbidity.
**Data S3.** Additional measures.
**Data S4.** Inferences by eyes.
**Figure S7.** Comparison of the overlap of the 95% confidence intervals of the standardized beta.
**Figure S8.** Comparison of the overlap of the 95% confidence intervals of the standardized beta coefficients on the association between affective responses and remembered pleasure.
**Data S5.** Correlation matrix.
**Table S2.** Correlation matrix with imputed data.
**Data S6.** Serial mediation models.
**Figure S9.** Serial mediation models.
**Table S3.** Multiple mediation effect through remembered pleasure, forecasted pleasure and affective attitudes in the association between affective response and subsequent daily time spent in MVPA.
**Table S4.** Multiple mediation effect through self‐efficacy towards exercise and towards MVPA in the association between the last affective response and subsequent daily time spent in MVPA.
**Table S5.** Multiple mediation effect through remembered pleasure, forecasted pleasure and affective Attitudes in the association between affective response and subsequent daily time spent in total PA.
**Table S6.** Multiple mediation effect through self‐efficacy towards exercise and towards MVPA in the association between the last affective response and subsequent daily time spent in total PA.
**Data S7.** Additional descriptive analyses.
**Figure S10.** Affective responses and perceived exertion during the exercise session.

## Data Availability

Deidentified data, data management, analysis codes and research materials have been made publicly available on Zenodo (https://doi.org/10.5281/zenodo.19387250).

## Footnotes

^1^
Some variables in the online questionnaire and the two reaction time tasks (i.e., the Single Category Implicit Association Test and the Manikin Task) belong to another project associated with the same ethics number and are not relevant to the present study.

^2^
Although the AHBF models a complete reflective processing chain that includes behavioural intentions as a proximal determinant of behaviour (Stevens et al., 2020; Williams & Evans, 2014), behavioural intention was not included as a mediator in *EA*_2_ due to statistical power constraints—our model already included three sequential mediators. Exploratory analyses nonetheless showed that affective attitudes predicted intentions towards MVPA (*β* = .39, *p* < .001), but that intentions predicted daily MVPA only when confounders were not controlled for (*β* = .30, *p* = .011 vs. *β* = .19, *p* = .119) and did not predict daily total PA either with or without confounders (*p* > .05). These findings suggest that the overall pattern of results is not substantially altered by the omission of intentions from the model. Detailed results are reported in Supplementary Material 6 (*R outputs for Affective Attitudes ➔ Intentions*)’.
