# Supplementary material for: Unravelling the relationship between exercise‐induced affective responses and daily physical activity in people with chronic diseases: Spillover or confounding effect?
Source: Br J Health Psychol. 2026 Jul 24;31(3):e70097. doi: 10.1111/bjhp.70097 (PMC13399999; doi:10.1111/bjhp.70097)
Supplement: Supplementary file 1 — Data S1. Sample size calculation and sensitivity analyses. Figure S1. A priori power analysis for H 1 and H 2. Figure S2. Univariable regression analyses. Figure S3. Association between affective responses and daily MVPA controlling for confounders. Figure S4. Mediation analyses (path b and c'). Data S2. Exercise sessions and participants information. Table S1. Exercise sessions' information. Figure S5. Exercise sessions' characteristics. Figure S6. Co‐occurrence between chronic conditions in participants with multimorbidity. Data S3. Additional measures. Data S4. Inferences by eyes. Figure S7. Comparison of the overlap of the 95% confidence intervals of the standardized beta. Figure S8. Comparison of the overlap of the 95% confidence intervals of the standardized beta coefficients on the association between affective responses and remembered pleasure. Data S5. Correlation matrix. Table S2. Correlation matrix with imputed data. Data S6. Serial mediation models. Figure S9. Serial mediation models. Table S3. Multiple mediation effect through remembered pleasure, forecasted pleasure and affective attitudes in the association between affective response and subsequent daily time spent in MVPA. Table S4. Multiple mediation effect through self‐efficacy towards exercise and towards MVPA in the association between the last affective response and subsequent daily time spent in MVPA. Table S5. Multiple mediation effect through remembered pleasure, forecasted pleasure and affective Attitudes in the association between affective response and subsequent daily time spent in total PA. Table S6. Multiple mediation effect through self‐efficacy towards exercise and towards MVPA in the association between the last affective response and subsequent daily time spent in total PA. Data S7. Additional descriptive analyses. Figure S10. Affective responses and perceived exertion during the exercise session. [file BJHP-31-0-s001.docx]

**Unravelling the Relationship Between Exercise-Induced Affective Responses and Daily Physical Activity in People with Chronic Diseases:**

**Spillover or Confounding effect?**

**Supplementary Materials**

**Supplementary Material 1: Sample Size Calculation and Sensitivity Analyses**

**Supplementary Material 2. Exercise Sessions and Participants Information**

**Supplementary Material 3. Additional Measures**

**Supplementary Material 4. Inferences by Eyes**

**Supplementary Material 5: Correlation Matrix**

**Supplementary Material 6. Serial Mediation Models**

**Supplementary Material 7: Additional Descriptive Analyses**

**Supplementary Material 1: Sample Size Calculation and Sensitivity Analyses**

**Figure S1**

*A priori power analysis for H_1_ and H_2_*

*
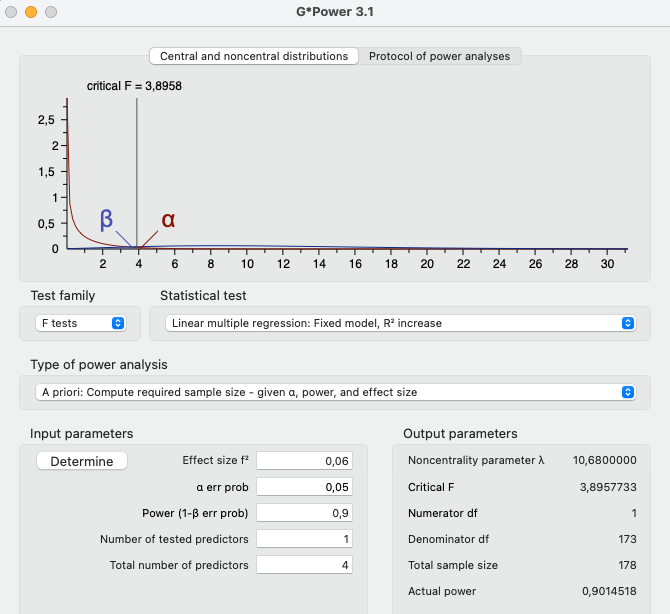
*

**Sensitive Power Analyses**

**Figure S2**

*
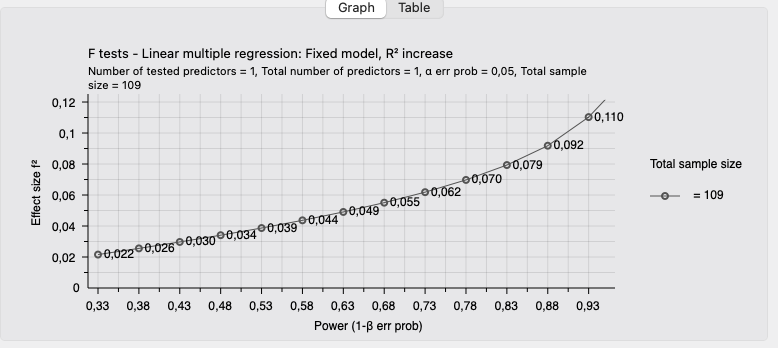
Univariable Regression Analyses*

*Note.* With an alpha level of .05 and statistical power ranging from 33% to 90%, the sensitivity power plot showed that the study could detect effect sizes of *f*^2^ = .02 at 33% power and *f*^2^ = .10 at 90% power.

**Figure S3**

*
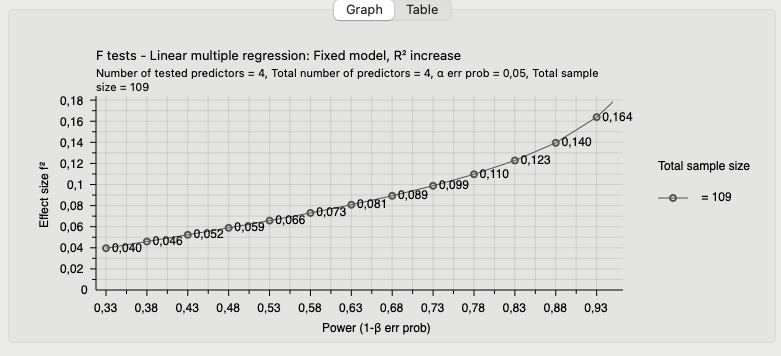
Association Between Affective Responses and Daily MVPA Controlling for Confounders*

*Note.* With an alpha level of .05 and statistical power ranging from 33% to 90%, the sensitivity power plot showed that the study could detect effect sizes of *f*^2^ = .04 at 33% power and *f*^2^ = .16 at 90% power.

**Figure S4**

*
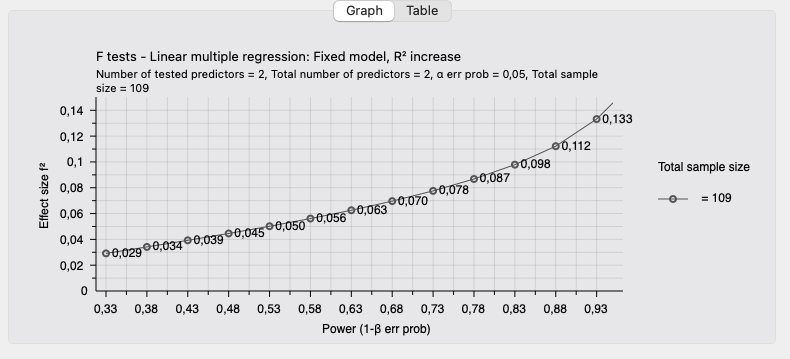
 Mediation Analyses (path b and c’)*

*Note.* With an alpha level of .05 and statistical power ranging from 33% to 90%, the sensitivity power plot showed that the study could detect effect sizes of *f*^2^ = .03 at 33% power and *f*^2^ = .13 at 90% power.

**Supplementary Material 2. Exercise Sessions and Participants Information**

**Table S1**

Exercise Sessions Information

| N = 109 | Number of participants (%) | Missing (%) |
| --- | --- | --- |
| Type of exercise |  | 7 (6.42) |
| Aqua-aerobic | 4 (3.92%) |  |
| Cardiovascular training | 5 (4.90%) |  |
| Circuit training | 30 (29.41%) |  |
| Core training | 1 (0.98%) |  |
| Hiking | 3 (2.94%) |  |
| Resistance training | 7 (6.86%) |  |
| Soft gym | 35 (34.31%) |  |
| Step workout | 1 (0.98%) |  |
| Walk | 9 (8.82%) |  |
| Walk and resistance training | 3 (2.94%) |  |
| Yoga | 4 (3.92%) |  |
| Session’s characteristics |  | 7 (6.42) |
| Intervals | 87 (85.29%) |  |
| Continue | 15 (14.71%) |  |
| Group | 102 (100%) |  |
| Individual | 0 (0%) |  |
| Indoor | 82 (80.39%) |  |
| Outdoor | 20 (19.61%) |  |
| Self-selected intensity | 20 (19.61%) |  |
| With music | 86 (84.31%) |  |
| Without music | 16 (15.69%) |  |
| With cool-down | 4 (3.92%) |  |
| Without cool-down | 98 (96.08%) |  |
| Exercise-sessions intensity |  | 9 (8.26) |
| Light (RPE_mean_<4) | 89 (89%) |  |
| Moderate (RPE_mean_ 4–5) | 7 (7%) |  |
| High (RPE_mean_ >5) | 4 (4%) |  |

*Note.* PA = physical activity. Soft gym, which involves low-impact, gentle movements aimed at improving flexibility, strength, and overall physical well-being, was the most common activity (34.31%). Light intensity was the most common exercise intensity (89%). Only four participants included a cool-down in their exercise sessions. Non-imputed data were carried out for the descriptive analyses.

**Figure S5**

*
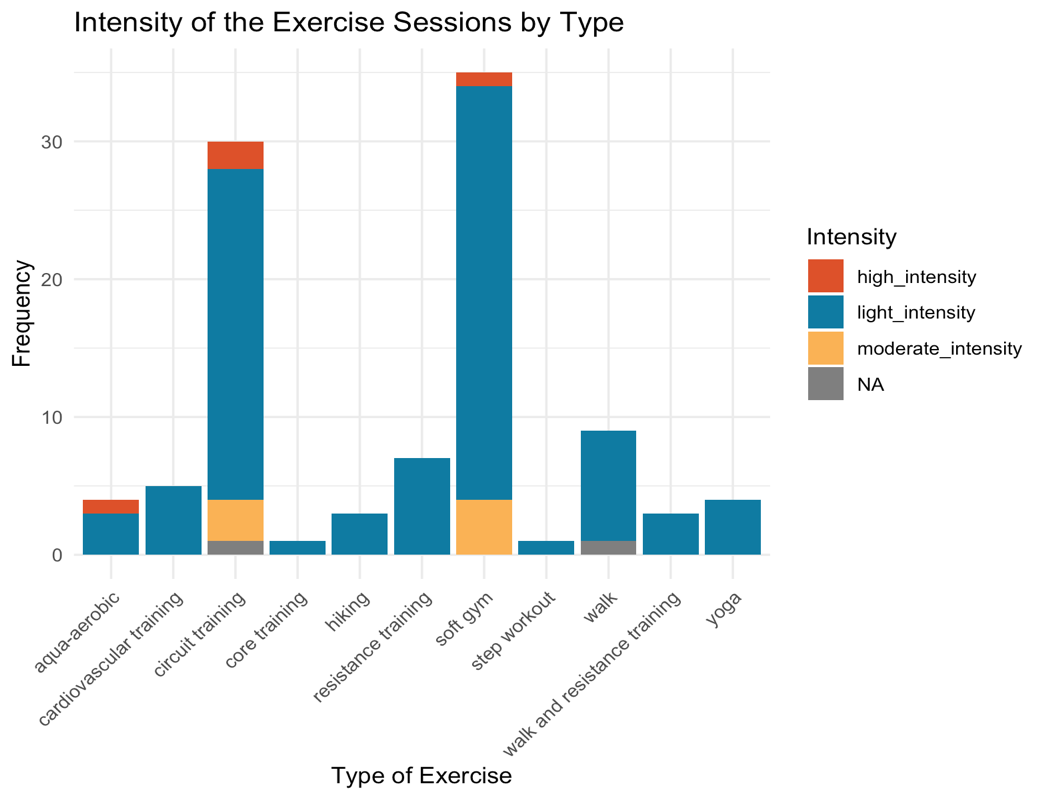
*Exercise Sessions Characteristics

A

*
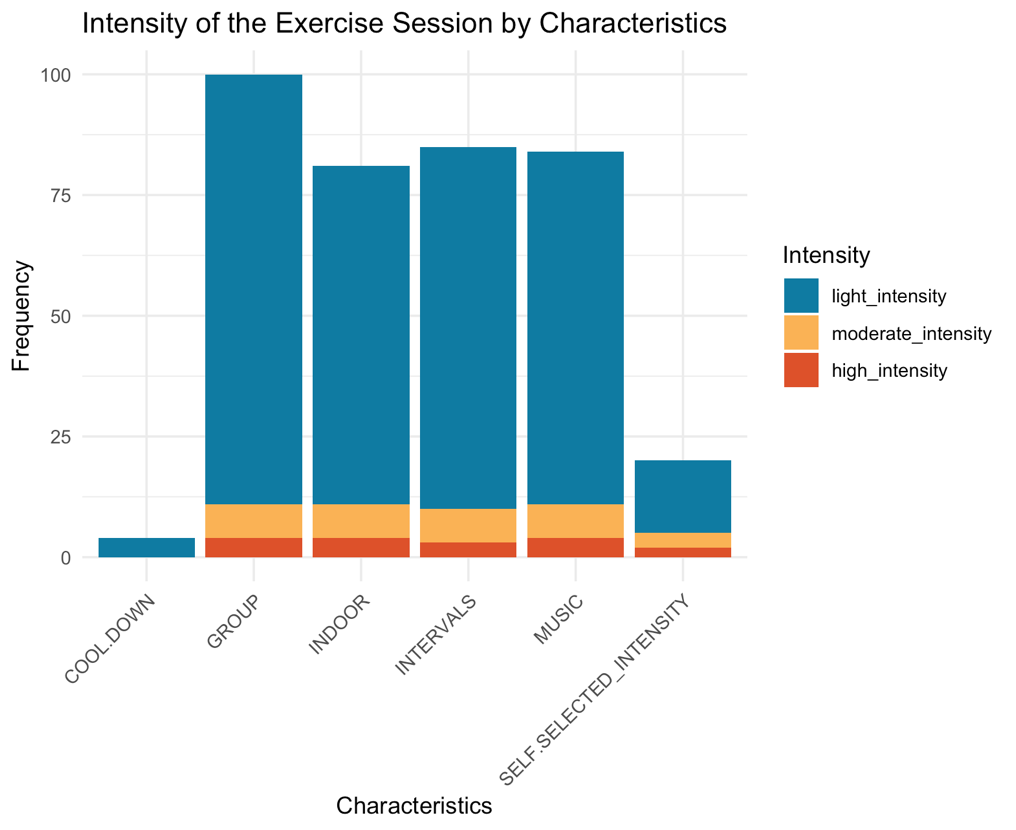
*

B

**Figure S6**


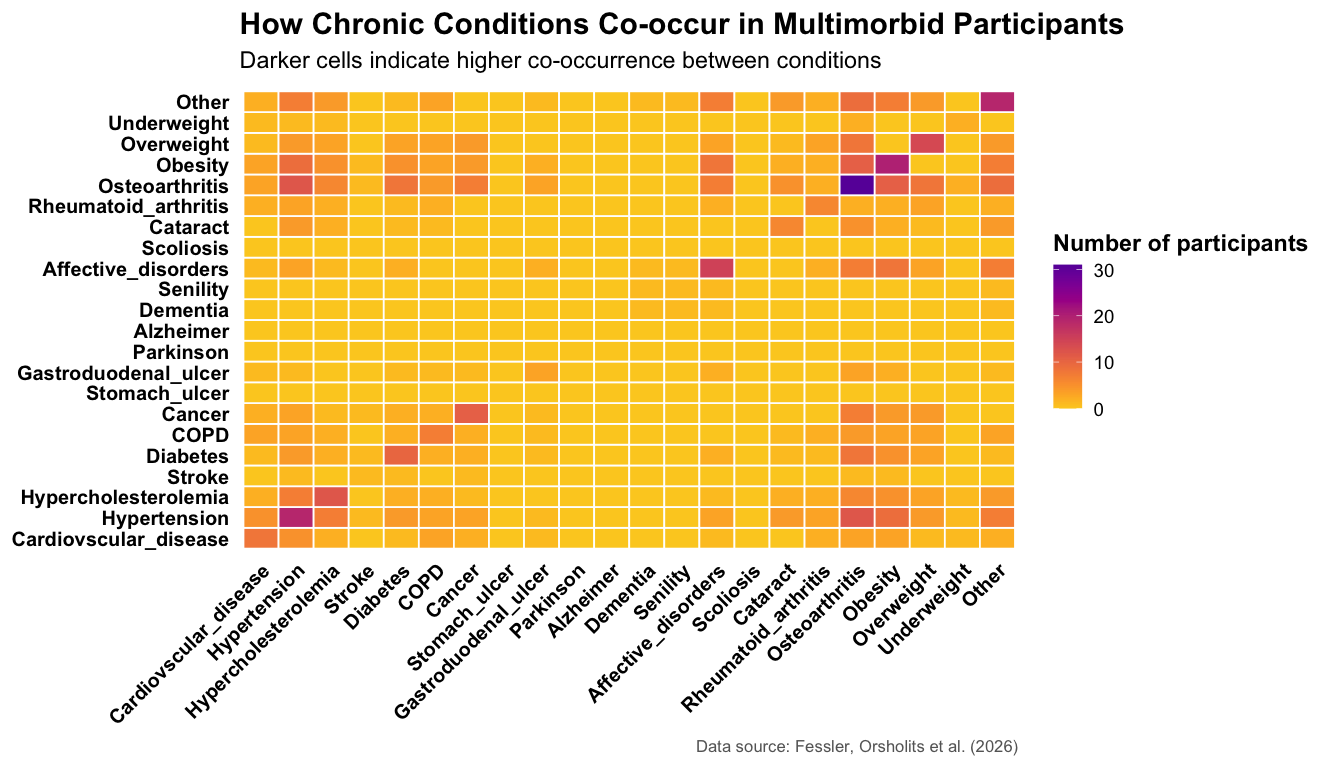
Co-occurrence Between Chronic Conditions in Participants with Multimorbidity

*Note*. Each cell represents the number of participants presenting with both conditions simultaneously. Darker cells indicate higher co-occurrence. Diagonal cells represent the number of participants reporting each condition. The predominantly light colouring of off-diagonal cells reflects the very small number of participants sharing any given pair of conditions—often fewer than five—illustrating the complexity and dispersion of multimorbidity profiles in the present sample, and the impracticality of conducting subgroup analyses by chronic condition. COPD = chronic obstructive pulmonary disease*.*

**Supplementary Material 3. Additional Measures**

**Post-Exercise Measure**

Global perceived exertion was measured using the following item: “Overall, what level of effort did you put in during the exercise session?” (Foster et al., 2001). Participants answered on a scale from 0 (*no effort at all*) to 10 (*maximal effort*).

Intention to engage in PA over the following week was measured using the item: “Over the next 7 days, I intend to do at least 30 minutes of MVPA a day on most days of the week during my free time or when travelling.” (Cheval et al., 2015). Participants answered on a 7-point Likert scale ranging from 1 (*strongly disagree*) to 7 (*strongly agree*).

**Participants Characteristics**

The socioeconomic status of the participants was evaluated by inquiring about their primary, secondary, and tertiary education qualifications, as well as their financial comfort (Fessler et al., 2023).

Instrumental attitudes towards PA were assessed using the following item “For me, doing at least 30 minutes of MVPA a day on most days of the week in my free time or when travelling is something…” (Phipps et al., 2021). Participants answered on two scales from “1 (*useless, harmful*)” to “7 (*useful, beneficial*)”. Items were averaged (Pearson correlation =.73).

Additional measures were taken 24-48 hours after the exercise session. These measures belong to another associated project and are not relevant to the present study. The additional measures included motivations towards physical activity (Motivation Scale Towards Health-Oriented Physical Activity; Boiché et al., 2019), automatic affective evaluation towards physical activity (Single-Category Implicit Association Test; Karpinski & Steinman, 2006) and approach-avoidance tendencies towards physical activity (Conflictual Manikin Task; Maltagliati, 2023).

**Supplementary Material 4. Inferences by Eyes**

This method states that when comparing two independent means, *p* <.05 if the *proportion of overlap* (i.e., the vertical distance between the upper CI of the lowest mean and the lower CI of the highest mean) of the 95% CIs is lower than 0.50 (Cumming & Finch, 2005). Specifically, we compared the *β* and its 95% CI of the four univariable linear regressions (four measures) two by two. To do this, we first computed the *margin of error* for each *β* as follows: $\frac{Upper CI-Lower CI}{2}$. Then, we computed the *average margin of error* as follows: $\frac{Margin of error model 1 + Margin of error model 2}{2}$. The *overlap* between the upper CI of the lowest *β* and the lower CI of the highest *β* were then computed as follows: $upper CI of the lowest \beta- lower CI of the highest \beta$. Finally, we computed the *proportion overlap* as follows: $\frac{Overlap}{Average margin of error}$.

**Figure S7**

Comparison of the Overlap of the 95% Confidence Intervals of the Standardised Beta Coefficients on the Association Between Affective Responses and Daily MVPA

*Note.* w = margin of error. Affective responses were measured four times during an exercise session: at the beginning of the warm-up (FS_1_), at the beginning (FS_2_) and at the end (FS_3_) of the workout, and at the end of the cool-down (FS_4_). Proportion overlap of the 95% confidence intervals (CIs) are all > 0.50, suggesting that the difference between the coefficient is not significant (*p* >.05).

**Figure S8**

Comparison of the Overlap of the 95% Confidence Intervals of the Standardised Beta Coefficients on the Association Between Affective Responses and Remembered Pleasure

*****Note.* FS = Feeling Scale; RP = remembered pleasure. Affective responses were measured four times during an exercise session: at the beginning of the warm-up (FS_1_), at the beginning (FS_2_) and at the end (FS_3_) of the workout, and at the end of the cool-down (FS_4_). Proportion overlap of the 95% confidence intervals (CIs) are all > 0.50, suggesting that the difference between the coefficient is not significant (*p* >.05).

**Supplementary Material 5: Correlation Matrix**

**Table S2**

Correlation Matrix with Imputed Data

| Variable 1 | Variable 2 | Correlation (r) | P-Value | lower95 | upper95 |
| --- | --- | --- | --- | --- | --- |
| Age | Gender | -0.0976991446 | 0.33296983868437413089935717 | -0.288043257 | 0.100071522 |
| Age | BMI | -0.3174841467 | 0.00106957667630595333087273 | -0.482207290 | -0.131078604 |
| Age | FS1 | 0.0533216633 | 0.60442761374486964065511074 | -0.147472799 | 0.249894826 |
| Age | FS2 | -0.0374556622 | 0.72765282104740991453439847 | -0.243389615 | 0.171709571 |
| Age | FS3 | 0.0623396262 | 0.53035675297642792536123579 | -0.131787813 | 0.251862033 |
| Age | FS4 | -0.1033569682 | 0.30642339130781198752728756 | -0.293604264 | 0.094765996 |
| Age | RPE1 | 0.1024693486 | 0.33054520587464936154376005 | -0.103925967 | 0.300403526 |
| Age | RPE2 | -0.0675418116 | 0.50272993042199809465842009 | -0.259402990 | 0.129448214 |
| Age | RPE3 | -0.1008737143 | 0.30542794651296151231534282 | -0.286536467 | 0.092091227 |
| Age | RPE4 | 0.0318807681 | 0.75476626193821028110164661 | -0.166648842 | 0.227926202 |
| Age | RP | 0.0015358957 | 0.99005176807057460042216235 | -0.235396682 | 0.238296157 |
| Age | FP | 0.1074040120 | 0.35898262999882180057653613 | -0.121943973 | 0.325863801 |
| Age | Intention towards PA | 0.1308527293 | 0.24596905059376686364380760 | -0.090473966 | 0.339862193 |
| Age | Self-efficacy towards exercise | -0.0172454390 | 0.86790952239399776768635775 | -0.216994064 | 0.183889317 |
| Age | Past MVPA | -0.0091016942 | 0.93555817211829439550285770 | -0.225782167 | 0.208436892 |
| Age | Self-efficacy towards PA | -0.1925367841 | 0.06310253517861295902147134 | -0.380452296 | 0.010647417 |
| Age | Affective attitudes towards PA | -0.0215567835 | 0.83637811217418378539889545 | -0.222387654 | 0.181028999 |
| Age | Instrumental attitudes towards PA | -0.2785772926 | 0.00557058109783164188966165 | -0.452987505 | -0.083629507 |
| Age | Daily MVPA | -0.0125382669 | 0.91139412230591443364602355 | -0.229238244 | 0.205345897 |
| Gender | BMI | -0.0647012616 | 0.52100154277036114791599175 | -0.256777842 | 0.132292213 |
| Gender | FS1 | 0.0474740125 | 0.64258768177337066695997692 | -0.151952001 | 0.243185831 |
| Gender | FS2 | 0.0682490446 | 0.52820874037407095080709496 | -0.143063130 | 0.273610125 |
| Gender | FS3 | 0.0949535065 | 0.34197292073713936311207817 | -0.100851279 | 0.283676824 |
| Gender | FS4 | 0.0481654241 | 0.62917156772770554962193046 | -0.146382978 | 0.239126696 |
| Gender | RPE1 | -0.0892129353 | 0.37876934772343967550156663 | -0.280883253 | 0.109300809 |
| Gender | RPE2 | -0.2970847918 | 0.00214115950633301382888263 | -0.463599064 | -0.110301718 |
| Gender | RPE3 | -0.1993004657 | 0.03833827811981900535176493 | -0.374078919 | -0.010850224 |
| Gender | RPE4 | 0.0534459760 | 0.59078751143886876207034220 | -0.140568917 | 0.243507952 |
| Gender | RP | 0.0961238701 | 0.36305865628051481319715776 | -0.110896086 | 0.295148933 |
| Gender | FP | 0.1172070954 | 0.26840881968052682227110495 | -0.090528742 | 0.315168677 |
| Gender | Intention towards PA | 0.0907329431 | 0.40198859595383801268653201 | -0.121195929 | 0.294750604 |
| Gender | Self-efficacy towards exercise | 0.0788261016 | 0.44560415957934168540433006 | -0.123347769 | 0.274717265 |
| Gender | Past MVPA | 0.0378135614 | 0.70803446900965028021346370 | -0.158807808 | 0.231549963 |
| Gender | Self-efficacy towards PA | -0.1339638976 | 0.22288221568407884376483707 | -0.337694235 | 0.081756737 |
| Gender | Affective attitudes towards PA | 0.1530593390 | 0.14513893813170972157955418 | -0.053216703 | 0.346807664 |
| Gender | Instrumental attitudes towards PA | 0.0566763302 | 0.59961618260964877347163338 | -0.153864986 | 0.262294665 |
| Gender | Daily MVPA | -0.1625490928 | 0.13933313245185846573903632 | -0.363963474 | 0.053388499 |
| BMI | FS1 | -0.2003679188 | 0.04191188870421505563435005 | -0.378922159 | -0.007431024 |
| BMI | FS2 | -0.1696614997 | 0.09528224855543887539255365 | -0.356266523 | 0.029957920 |
| BMI | FS3 | -0.3158184704 | 0.00118882298293839124003757 | -0.481326543 | -0.128566025 |
| BMI | FS4 | -0.3167989561 | 0.00099502110468015171271250 | -0.480336463 | -0.131973994 |
| BMI | RPE1 | 0.0671894676 | 0.49812804744807598344991106 | -0.126710310 | 0.256143522 |
| BMI | RPE2 | 0.3034813378 | 0.00178463023685107905445812 | 0.116224395 | 0.469904158 |
| BMI | RPE3 | 0.3461559426 | 0.00024048243142521665656128 | 0.166782540 | 0.503350999 |
| BMI | RPE4 | 0.2027401934 | 0.04038594288022928635584208 | 0.009008073 | 0.381803144 |
| BMI | RP | -0.2012878133 | 0.04565030976611435947765116 | -0.383538998 | -0.003946291 |
| BMI | FP | -0.1733772519 | 0.08898125697604433725906858 | -0.360083515 | 0.026682531 |
| BMI | Intention towards PA | -0.3053927745 | 0.00220533589824662996231597 | -0.475720062 | -0.112990372 |
| BMI | Self-efficacy towards exercise | -0.1236516372 | 0.22741019787319013967241688 | -0.315012421 | 0.077370038 |
| BMI | Past MVPA | -0.0453536640 | 0.66423328215378263372770107 | -0.245208894 | 0.158199248 |
| BMI | Self-efficacy towards PA | -0.0176311502 | 0.87171211848257934562411720 | -0.227591418 | 0.193895682 |
| BMI | Affective attitudes towards PA | -0.1972761108 | 0.05129066827070010575750914 | -0.380730695 | 0.001120399 |
| BMI | Instrumental attitudes towards PA | -0.0018159801 | 0.98550280881212803940627509 | -0.195160858 | 0.191664765 |
| BMI | Daily MVPA | -0.3665161278 | 0.00014939934711194160403894 | -0.524892821 | -0.183609690 |
| FS1 | FS2 | 0.4668726761 | 0.00000163482187998709753184 | 0.290549761 | 0.612530301 |
| FS1 | FS3 | 0.2552220670 | 0.00985241972097454336843025 | 0.062714190 | 0.429419797 |
| FS1 | FS4 | 0.1705412236 | 0.09611961637901297872410566 | -0.030627083 | 0.358431270 |
| FS1 | RPE1 | -0.1264169922 | 0.22689853671506585164330261 | -0.321432717 | 0.078885924 |
| FS1 | RPE2 | -0.1865050219 | 0.05855736778183896762417504 | -0.366399540 | 0.006830299 |
| FS1 | RPE3 | 0.0570361183 | 0.57646501762877699892584360 | -0.142286259 | 0.251913087 |
| FS1 | RPE4 | -0.0909713416 | 0.35337436935863775211430493 | -0.276489656 | 0.101083635 |
| FS1 | RP | 0.2965731030 | 0.00348872584815419946313586 | 0.100261554 | 0.470658968 |
| FS1 | FP | 0.2464288442 | 0.01407106111853318949900249 | 0.050727834 | 0.423905046 |
| FS1 | Intention towards PA | 0.0819361400 | 0.45527401843675630033203561 | -0.132659320 | 0.289193861 |
| FS1 | Self-efficacy towards exercise | 0.2489265669 | 0.01234554707670184464651797 | 0.055038031 | 0.424724696 |
| FS1 | Past MVPA | 0.0032441336 | 0.97595455773046302372364380 | -0.204774407 | 0.210982291 |
| FS1 | Self-efficacy towards PA | 0.1563719943 | 0.15100246838601705245963558 | -0.057465295 | 0.356490633 |
| FS1 | Affective attitudes towards PA | 0.1134119953 | 0.27558399224250007542025287 | -0.090604621 | 0.308292826 |
| FS1 | Instrumental attitudes towards PA | -0.0939847030 | 0.35381667223061608051892790 | -0.285369813 | 0.104608372 |
| FS1 | Daily MVPA | -0.0510959172 | 0.63523787143558607670712490 | -0.256575958 | 0.158803203 |
| FS2 | FS3 | 0.5751252283 | 0.00000000002815009653589475 | 0.431897850 | 0.690058907 |
| FS2 | FS4 | 0.4622800980 | 0.00000123250459119652232052 | 0.289554081 | 0.605847767 |
| FS2 | RPE1 | 0.0151572569 | 0.87887017663981226167635441 | -0.177874328 | 0.207065601 |
| FS2 | RPE2 | -0.2747838304 | 0.00455652717254534551927225 | -0.443746903 | -0.086955148 |
| FS2 | RPE3 | -0.1916760254 | 0.05642168412248971948175225 | -0.374342102 | 0.005310213 |
| FS2 | RPE4 | -0.2182114455 | 0.02911252673237653063043062 | -0.397764797 | -0.022560095 |
| FS2 | RP | 0.3689424418 | 0.00022925019778451301288857 | 0.179264533 | 0.532174546 |
| FS2 | FP | 0.3229159813 | 0.00190904178831351190037680 | 0.122812131 | 0.497789436 |
| FS2 | Intention towards PA | 0.1504560856 | 0.18935994595781333038608807 | -0.074653828 | 0.360975484 |
| FS2 | Self-efficacy towards exercise | 0.3176235393 | 0.00173896275341131113269244 | 0.122468278 | 0.489131008 |
| FS2 | Past MVPA | 0.0314641847 | 0.80083402591703289896685192 | -0.209896656 | 0.269210451 |
| FS2 | Self-efficacy towards PA | 0.3291544639 | 0.00297339938521845158908574 | 0.115780756 | 0.513489281 |
| FS2 | Affective attitudes towards PA | 0.2183377377 | 0.04025491578879683562952607 | 0.009862186 | 0.408623281 |
| FS2 | Instrumental attitudes towards PA | 0.0344761524 | 0.75584341800745435957509244 | -0.180896190 | 0.246693232 |
| FS2 | Daily MVPA | 0.1492848462 | 0.17165740087822728421329543 | -0.065166583 | 0.350555434 |
| FS3 | FS4 | 0.4951275616 | 0.00000008385313203831681145 | 0.331298249 | 0.629975273 |
| FS3 | RPE1 | 0.1241176434 | 0.21374539114693072439088439 | -0.071786000 | 0.310800804 |
| FS3 | RPE2 | -0.1814385539 | 0.07493306746181822852737042 | -0.367379124 | 0.018448198 |
| FS3 | RPE3 | -0.2873571734 | 0.00282234935912802596680304 | -0.454006572 | -0.101282254 |
| FS3 | RPE4 | -0.1381549345 | 0.16090149444129067668463051 | -0.321588860 | 0.055273605 |
| FS3 | RP | 0.5302028083 | 0.00000001820841198108042622 | 0.366883810 | 0.661815416 |
| FS3 | FP | 0.4416507334 | 0.00000251171447571959865694 | 0.269940957 | 0.586135923 |
| FS3 | Intention towards PA | 0.0969564986 | 0.35307731040979190018092027 | -0.107599286 | 0.293636726 |
| FS3 | Self-efficacy towards exercise | 0.2263688461 | 0.02421137546843711227384510 | 0.030019986 | 0.405896705 |
| FS3 | Past MVPA | -0.0960680710 | 0.37414402784302680515793327 | -0.299420469 | 0.115632814 |
| FS3 | Self-efficacy towards PA | 0.1577923806 | 0.13157152379074374404233083 | -0.047663525 | 0.350438412 |
| FS3 | Affective attitudes towards PA | 0.1809276136 | 0.08235895942815235626355275 | -0.023459355 | 0.370796903 |
| FS3 | Instrumental attitudes towards PA | -0.0270008611 | 0.78790111917119742823700790 | -0.220098280 | 0.168132817 |
| FS3 | Daily MVPA | 0.1536380182 | 0.16510519218128796858913176 | -0.063713232 | 0.357073961 |
| FS4 | RPE1 | -0.1798932424 | 0.06620223109260611749071757 | -0.359153146 | 0.012167976 |
| FS4 | RPE2 | -0.2929632088 | 0.00220048612874512890258405 | -0.458178572 | -0.108180765 |
| FS4 | RPE3 | -0.3233299260 | 0.00074199875444857783628183 | -0.485532621 | -0.139606704 |
| FS4 | RPE4 | -0.4640141372 | 0.00000042517459667886412188 | -0.602539945 | -0.298347109 |
| FS4 | RP | 0.4117176369 | 0.00001666707673863290911100 | 0.234014922 | 0.562800529 |
| FS4 | FP | 0.3077485157 | 0.00263191071349058941866716 | 0.110347504 | 0.481791142 |
| FS4 | Intention towards PA | 0.1728922487 | 0.11001594064693570595991901 | -0.039522407 | 0.370356329 |
| FS4 | Self-efficacy towards exercise | 0.3429833266 | 0.00081523497998003643157799 | 0.147100250 | 0.512978882 |
| FS4 | Past MVPA | 0.2039530735 | 0.03882164928997317021597269 | 0.010623097 | 0.382584669 |
| FS4 | Self-efficacy towards PA | 0.2764082878 | 0.01124776962434419387559803 | 0.064277397 | 0.464639434 |
| FS4 | Affective attitudes towards PA | 0.3569501046 | 0.00041358801545590272337460 | 0.164632596 | 0.523121661 |
| FS4 | Instrumental attitudes towards PA | 0.2214445401 | 0.02755098302925685846420656 | 0.024892719 | 0.401512979 |
| FS4 | Daily MVPA | 0.2394325517 | 0.02147129717631336351391091 | 0.036046361 | 0.423773129 |
| RPE1 | RPE2 | 0.3797107619 | 0.00005535600482775985859037 | 0.202564627 | 0.532793424 |
| RPE1 | RPE3 | 0.2435220080 | 0.01284073990673140382157147 | 0.052706450 | 0.417181031 |
| RPE1 | RPE4 | 0.3242269379 | 0.00072654753772555301719049 | 0.140346570 | 0.486486957 |
| RPE1 | RP | -0.1254353148 | 0.20591805815278427238546044 | -0.310858455 | 0.069185778 |
| RPE1 | FP | -0.0008532989 | 0.99320624789860734171753620 | -0.194748569 | 0.193106152 |
| RPE1 | Intention towards PA | -0.0611721557 | 0.58779888783854294942443630 | -0.275422879 | 0.158868004 |
| RPE1 | Self-efficacy towards exercise | -0.2003009609 | 0.05889769440544904283152405 | -0.391630829 | 0.007632557 |
| RPE1 | Past MVPA | 0.0059778517 | 0.95580697732590458315371507 | -0.202607407 | 0.214044220 |
| RPE1 | Self-efficacy towards PA | 0.0063794268 | 0.95330345339993882713969242 | -0.204229852 | 0.216424264 |
| RPE1 | Affective attitudes towards PA | -0.0188003820 | 0.85651457625340210455533452 | -0.219011889 | 0.182930312 |
| RPE1 | Instrumental attitudes towards PA | -0.0065264303 | 0.95004240331907052752313803 | -0.207629715 | 0.195106157 |
| RPE1 | Daily MVPA | -0.0449471488 | 0.67627030605655846784429741 | -0.250640268 | 0.164629012 |
| RPE2 | RPE3 | 0.6315597819 | 0.00000000000006479350713291 | 0.500164050 | 0.734526973 |
| RPE2 | RPE4 | 0.3553998017 | 0.00017720504353780776687390 | 0.175522010 | 0.512314495 |
| RPE2 | RP | -0.2543878177 | 0.01117117704424699584087044 | -0.430910906 | -0.059113578 |
| RPE2 | FP | -0.2093389781 | 0.03951237786869803841893756 | -0.392505472 | -0.010201138 |
| RPE2 | Intention towards PA | -0.2920914686 | 0.00363050101891571313367235 | -0.464928198 | -0.097808606 |
| RPE2 | Self-efficacy towards exercise | -0.2905201250 | 0.00398348519212680333284604 | -0.464273751 | -0.095233321 |
| RPE2 | Past MVPA | -0.0429098936 | 0.67550601358254969142080881 | -0.239242319 | 0.156793584 |
| RPE2 | Self-efficacy towards PA | -0.1017008856 | 0.31365285608863291422210295 | -0.291833428 | 0.096163706 |
| RPE2 | Affective attitudes towards PA | -0.1592157069 | 0.10819662285072301211030776 | -0.342139152 | 0.035334767 |
| RPE2 | Instrumental attitudes towards PA | 0.0278064181 | 0.78608181347482120049363630 | -0.171338886 | 0.224768670 |
| RPE2 | Daily MVPA | -0.1483672097 | 0.19549827721441956351888791 | -0.359038852 | 0.076690240 |
| RPE3 | RPE4 | 0.4414008802 | 0.00000239720149451379940445 | 0.270141811 | 0.585585970 |
| RPE3 | RP | -0.1495232810 | 0.15125080420857309193571894 | -0.342039465 | 0.055039149 |
| RPE3 | FP | -0.0620322207 | 0.55640891839626682724428974 | -0.262771704 | 0.143854605 |
| RPE3 | Intention towards PA | -0.2688663870 | 0.01946459607200633559753733 | -0.467495927 | -0.044392637 |
| RPE3 | Self-efficacy towards exercise | -0.2459169201 | 0.02128526973966388818881867 | -0.433938656 | -0.037368170 |
| RPE3 | Past MVPA | 0.0406105982 | 0.68835062256696499805030953 | -0.156621735 | 0.234728139 |
| RPE3 | Self-efficacy towards PA | 0.0518689366 | 0.63659917320806080898165646 | -0.162012238 | 0.261095360 |
| RPE3 | Affective attitudes towards PA | -0.0348555082 | 0.73370295665812834684516019 | -0.231485617 | 0.164510694 |
| RPE3 | Instrumental attitudes towards PA | 0.1116334603 | 0.27886192325044090178920442 | -0.090547910 | 0.304977084 |
| RPE3 | Daily MVPA | -0.2533091293 | 0.01254783723877493997744814 | -0.431917966 | -0.055581908 |
| RPE4 | RP | -0.1015644068 | 0.33542183539762948640827744 | -0.299789176 | 0.105067134 |
| RPE4 | FP | -0.0197088332 | 0.84784302405216971365575773 | -0.217531050 | 0.179668672 |
| RPE4 | Intention towards PA | -0.0162968330 | 0.88868800734068886804095655 | -0.239767818 | 0.208814237 |
| RPE4 | Self-efficacy towards exercise | -0.1379322255 | 0.26660498705979901457041592 | -0.365941649 | 0.105698714 |
| RPE4 | Past MVPA | 0.0558489377 | 0.58347241882137923507656296 | -0.142946624 | 0.250312908 |
| RPE4 | Self-efficacy towards PA | -0.0703432371 | 0.51176669682769826419388437 | -0.273781004 | 0.139119358 |
| RPE4 | Affective attitudes towards PA | -0.0703479708 | 0.49644856597469422254675919 | -0.266913344 | 0.131836824 |
| RPE4 | Instrumental attitudes towards PA | -0.0012803227 | 0.98991455416659690502712010 | -0.197182048 | 0.194719724 |
| RPE4 | Daily MVPA | -0.1688384207 | 0.12262998117279544363356081 | -0.368677397 | 0.045917751 |
| RP | FP | 0.7355662979 | 0.00000000000000000001923984 | 0.630213467 | 0.814322583 |
| RP | Intention towards PA | 0.2110769675 | 0.03931150623979837166466211 | 0.010496344 | 0.395328412 |
| RP | Self-efficacy towards exercise | 0.3913627060 | 0.00009198656097584933339847 | 0.203347758 | 0.551541572 |
| RP | Past MVPA | -0.0510155076 | 0.66025441878836244047334958 | -0.271729889 | 0.174797384 |
| RP | Self-efficacy towards PA | 0.3047998678 | 0.00407076821127983802528494 | 0.099683705 | 0.485067100 |
| RP | Affective attitudes towards PA | 0.3532677081 | 0.00054997207940399772587575 | 0.158411242 | 0.521638700 |
| RP | Instrumental attitudes towards PA | 0.0083675522 | 0.93727868048380102994343588 | -0.197422824 | 0.213451585 |
| RP | Daily MVPA | 0.0637125080 | 0.55193872451092218511803367 | -0.145371503 | 0.267349013 |
| FP | Intention towards PA | 0.3315775314 | 0.00107861530085052461334916 | 0.137132842 | 0.501416751 |
| FP | Self-efficacy towards exercise | 0.3689587941 | 0.00025712959277139038789806 | 0.177672352 | 0.533379408 |
| FP | Past MVPA | 0.0562256526 | 0.61070951841206244647963786 | -0.159058951 | 0.266405859 |
| FP | Self-efficacy towards PA | 0.3477532606 | 0.00153358691776708236029214 | 0.137518175 | 0.528005979 |
| FP | Affective attitudes towards PA | 0.4121694987 | 0.00004160877503959156551100 | 0.224747387 | 0.570182576 |
| FP | Instrumental attitudes towards PA | 0.0272900195 | 0.81231003358139675096794008 | -0.195471481 | 0.247373675 |
| FP | Daily MVPA | 0.2519235009 | 0.01273770479286074307212484 | 0.054833545 | 0.430118934 |
| Intention towards PA | Self-efficacy towards exercise | 0.4976411916 | 0.00000045220868110278859016 | 0.322115575 | 0.640089985 |
| Intention towards PA | Past MVPA | 0.2327642189 | 0.02117578156737300390521916 | 0.035463803 | 0.412600662 |
| Intention towards PA | Self-efficacy towards PA | 0.2634746588 | 0.03106192921849356899932637 | 0.024562026 | 0.473917292 |
| Intention towards PA | Affective attitudes towards PA | 0.4172399448 | 0.00002887541563734236774500 | 0.231811973 | 0.573409300 |
| Intention towards PA | Instrumental attitudes towards PA | 0.2406664908 | 0.02277418890274294713260694 | 0.034184769 | 0.427443790 |
| Intention towards PA | Daily MVPA | 0.3116988362 | 0.00314088663078278609863681 | 0.108045749 | 0.490244972 |
| Self-efficacy towards exercise | Past MVPA | 0.0567539835 | 0.58869661348651880494742272 | -0.148026317 | 0.256867769 |
| Self-efficacy towards exercise | Self-efficacy towards PA | 0.2389936289 | 0.02138118813072414914344677 | 0.036121198 | 0.422947388 |
| Self-efficacy towards exercise | Affective attitudes towards PA | 0.2277758012 | 0.02663528280727227651181188 | 0.026856535 | 0.411006416 |
| Self-efficacy towards exercise | Instrumental attitudes towards PA | 0.1204004930 | 0.25873487442371695399501164 | -0.088742939 | 0.319375744 |
| Self-efficacy towards exercise | Daily MVPA | 0.2100306303 | 0.04826788425291366618763433 | 0.001623402 | 0.400952503 |
| Past MVPA | Self-efficacy towards PA | 0.1612407609 | 0.11353653141911713986278443 | -0.038779498 | 0.348837504 |
| Past MVPA | Affective attitudes towards PA | 0.1501233437 | 0.14513548449559582342871522 | -0.052180255 | 0.340591002 |
| Past MVPA | Instrumental attitudes towards PA | 0.1078922906 | 0.27024909595501322989719029 | -0.084043781 | 0.292108833 |
| Past MVPA | Daily MVPA | 0.0983533432 | 0.39492082989064924980482374 | -0.127950572 | 0.314922411 |
| Self-efficacy towards PA | Affective attitudes towards PA | 0.5503281730 | 0.00000146194080485351563961 | 0.351380210 | 0.701722684 |
| Self-efficacy towards PA | Instrumental attitudes towards PA | 0.2647220792 | 0.01933394243976536280849032 | 0.043919299 | 0.460867012 |
| Self-efficacy towards PA | Daily MVPA | 0.2714026790 | 0.00713622946564974793698388 | 0.075436552 | 0.447183892 |
| Affective attitudes towards PA | Instrumental attitudes towards PA | 0.4470434653 | 0.00000539852355380347370304 | 0.267104161 | 0.596860112 |
| Affective attitudes towards PA | Daily MVPA | 0.1604392590 | 0.12354634620857515847625052 | -0.044097420 | 0.352066856 |
| Instrumental attitudes towards PA | Daily MVPA | 0.0616116061 | 0.61084708811475940670732143 | -0.174124494 | 0.290668667 |

*Note.* BMI = body mass index; PA = physical activity; MVPA = moderate-to-vigorous physical activity; FS = feeling scale; RPE = rating of perceived exertion. N = 109.

**Supplementary Material 6. Serial Mediation Models**

**Affective Processing Pathway**

Serial mediation models were computed to test the pathway from affective responses (ARs) to remembered pleasure, then to forecasted pleasure, followed by affective attitudes, and finally to daily MVPA, while controlling for confounders (i.e., BMI, perceived exertion; Figure S9). In this model, paths $a_{1}$ to $a_{6}$ represent the direct effects of ARs on remembered pleasure ($a_{1}$), remembered pleasure on forecasted pleasure ($a_{2}$), ARs on forecasted pleasure ($a_{3}$), forecasted pleasure on affective attitudes ($a_{4}$), remembered pleasure on affective attitudes ($a_{5}$), and ARs on affective attitudes ($a_{6}$), all adjusted for confounders. Paths $b_{1}$, $b_{2}$, and $b_{3}$represent the effects of affective attitudes, forecasted pleasure, and remembered pleasure on daily MVPA, respectively, while controlling for ARs, other mediators in the model, and confounders. Two specific indirect pathways were estimated: the indirect effect of ARs on daily MVPA via forecasted pleasure and affective attitudes was quantified as the product $a_{3}\times a_{4}\times b_{1}$, and the indirect effect via affective attitudes alone was quantified as $a_{6}\times b_{1}$. The total indirect effect was computed as the sum of these two pathways ($a_{3}\times a_{4}\times b_{1}+a_{6}\times b_{1}$), and the total effect of ARs on daily MVPA was calculated as the sum of the direct effect ($c^{'}$) and this total indirect effect.

**Self-Efficacy Pathway**

Serial mediation models were also computed to test the pathway from ARs to session-specific self-efficacy, then to MVPA-specific self-efficacy, and finally to daily MVPA, while controlling for the same confounders (Figure S9). Paths $a_{1}$ to $a_{3}$ represent the direct effects of ARs on session-specific self-efficacy ($a_{1}$), session-specific self-efficacy on self-efficacy towards MVPA ($a_{2}$), and ARs on self-efficacy towards MVPA ($a_{3}$). Path $b_{1}$ represents the effect of self-efficacy towards MVPA on daily MVPA controlling for ARs, session-specific self-efficacy, and confounders, while path $b_{2}$ represents the effect of session-specific self-efficacy on daily MVPA controlling for ARs and confounders. Two distinct indirect effects were estimated for this pathway: the indirect effect of ARs on daily MVPA via session-specific self-efficacy was quantified as $a_{1}\times b_{2}$, and the indirect effect via MVPA-specific self-efficacy was quantified as $a_{3}\times b_{1}$. The total indirect effect was computed as the sum of these two pathways ($a_{1}\times b_{2}+a_{3}\times b_{1}$). The total effect of ARs on daily MVPA was then derived by summing the direct effect ($c^{'}$) and the total indirect effect.

**Figure S9**

Serial Mediation Models

*Note.* MVPA = moderate-to-vigorous physical activity.

**1. Affective Pathway (MVPA)**

**R outputs** **for Affective Responses 🡪 Remembered Pleasure**


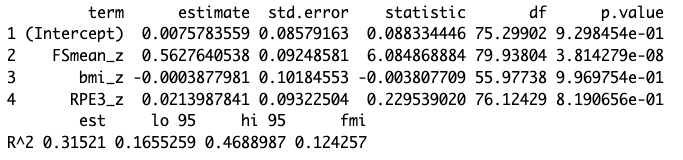
**FSmean**

*Note.* FSmean = average affective response; bmi = body mass index; RPE3 = third perceived exertion score. All scored were standardised (z-score).

**
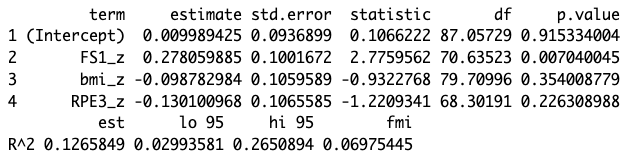
FS1**

*Note.* FS1 = first affective response; bmi = body mass index; RPE3 = third perceived exertion score. All scored were standardised (z-score).

**
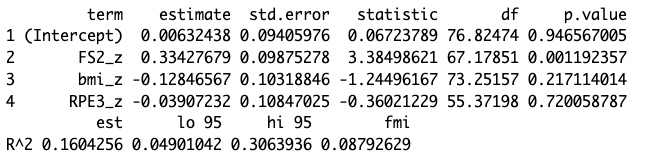
FS2**

*Note.* FS2 = second affective response; bmi = body mass index; RPE3 = third perceived exertion score. All scored were standardised (z-score).

**
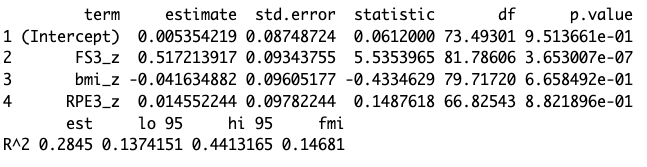
FS3**

*Note.* FS3 = third affective response; bmi = body mass index; RPE3 = third perceived exertion score. All scored were standardised (z-score).

**
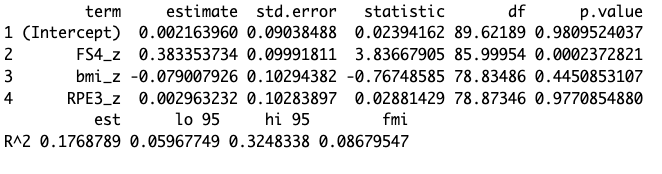
FS4**

*Note.* FS4 = last affective response; bmi = body mass index; RPE3 = third perceived exertion score. All scored were standardised (z-score).

**R outputs** **for Affective Responses 🡪 Remembered Pleasure 🡪 Forecasted Pleasure**

**
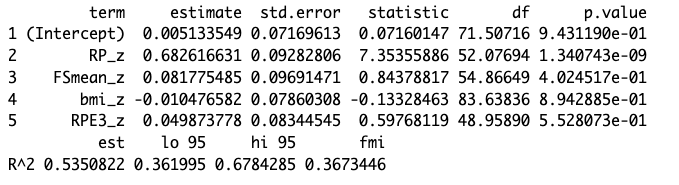
FSmean**

*Note.* FSmean = average affective response; bmi = body mass index; RPE3 = third perceived exertion score; RP = remembered pleasure. All scored were standardised (z-score).

**
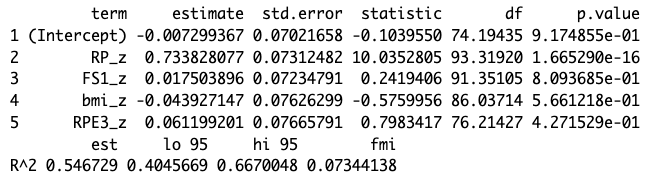
FS1**

*Note.* FS1 = first affective response; bmi = body mass index; RPE3 = third perceived exertion score; RP = remembered pleasure. All scored were standardised (z-score).

**
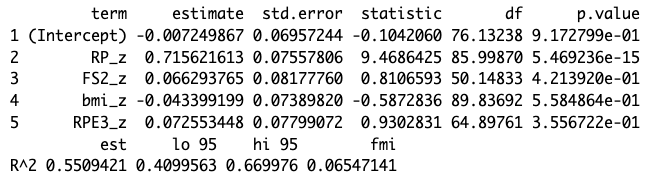
FS2**

*Note.* FS2 = second affective response; bmi = body mass index; RPE3 = third perceived exertion score; RP = remembered pleasure. All scored were standardised (z-score).

**
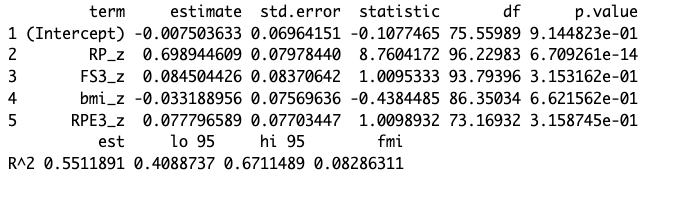
FS3**

*Note.* FS3 = third affective response; bmi = body mass index; RPE3 = third perceived exertion score; RP = remembered pleasure. All scored were standardised (z-score).

*
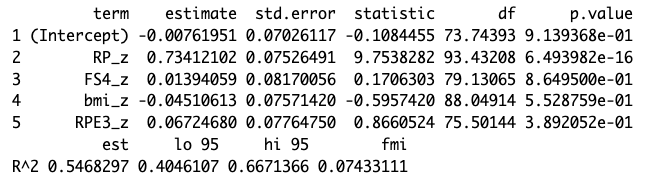
***FS4**

*Note.* FS4 = last affective response; bmi = body mass index; RPE3 = third perceived exertion score; RP = remembered pleasure. All scored were standardised (z-score).

**R outputs** **for Affective Responses 🡪 Remembered Pleasure 🡪 Forecasted Pleasure 🡪 Affective Attitudes**

**
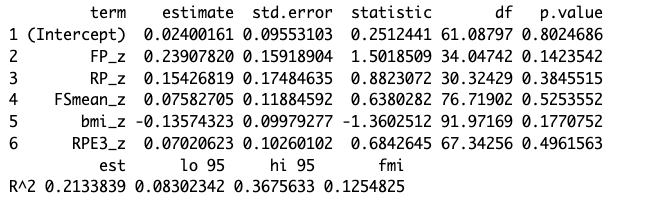
FSmean**

*Note.* FSmean = average affective response; bmi = body mass index; RPE3 = third perceived exertion score; RP = remembered pleasure; FP = forecasted pleasure. All scored were standardised (z-score).

**
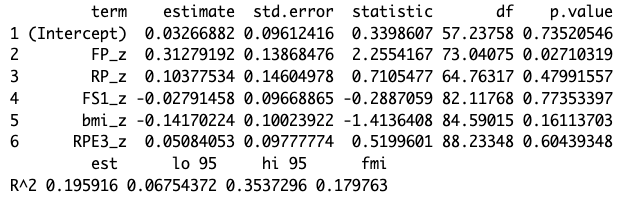
FS1**

*Note.* FS1 = first affective response; bmi = body mass index; RPE3 = third perceived exertion score; RP = remembered pleasure; FP = forecasted pleasure. All scored were standardised (z-score).

**
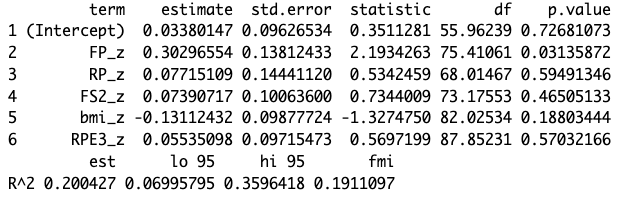
FS2**

*Note.* FS2 = second affective response; bmi = body mass index; RPE3 = third perceived exertion score; RP = remembered pleasure; FP = forecasted pleasure. All scored were standardised (z-score).

**
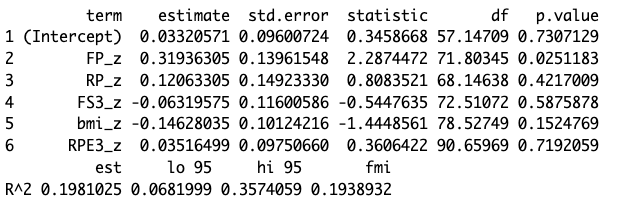
FS3**

*Note.* FS3 = third affective response; bmi = body mass index; RPE3 = third perceived exertion score; RP = remembered pleasure; FP = forecasted pleasure. All scored were standardised (z-score).

**
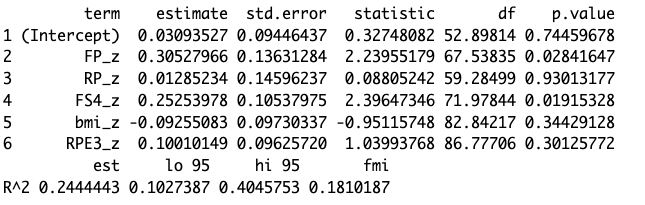
FS4**

*Note.* FS4 = last affective response; bmi = body mass index; RPE3 = third perceived exertion score; RP = remembered pleasure; FP = forecasted pleasure. All scored were standardised (z-score).

**R outputs** **for Affective Responses 🡪 Forecasted Pleasure 🡪 Affective Attitudes 🡪 Daily MVPA**

**
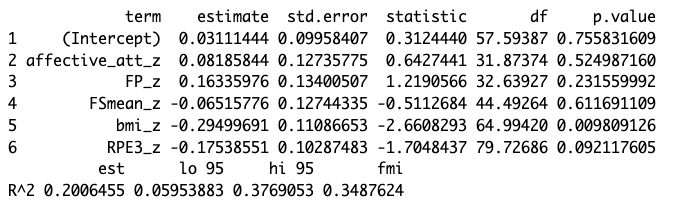
FSmean**

*Note.* FSmean = average affective response; bmi = body mass index; RPE3 = third perceived exertion score; RP = remembered pleasure; FP = forecasted pleasure; affective_att = affective attitudes. All scored were standardised (z-score).

*
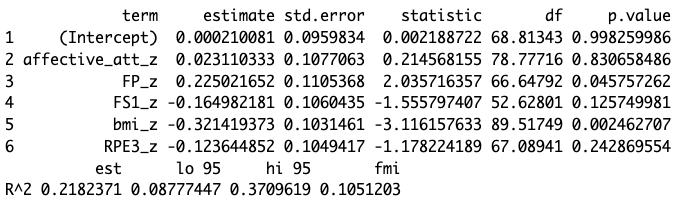
***FS1**

*Note.* FS1 = first affective response; bmi = body mass index; RPE3 = third perceived exertion score; RP = remembered pleasure; FP = forecasted pleasure; affective_att = affective attitudes. All scored were standardised (z-score).

*
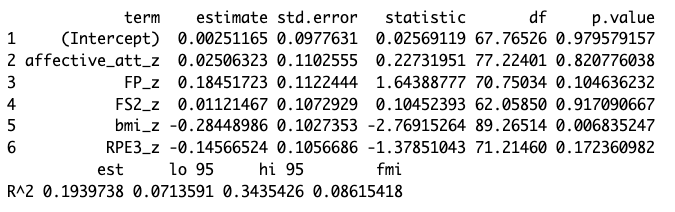
***FS2**

*Note.* FS2 = second affective response; bmi = body mass index; RPE3 = third perceived exertion score; RP = remembered pleasure; FP = forecasted pleasure; affective_att = affective attitudes. All scored were standardised (z-score).


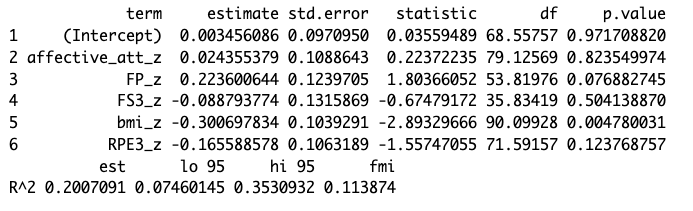
**FS3**

*Note.* FS3 = third affective response; bmi = body mass index; RPE3 = third perceived exertion score; RP = remembered pleasure; FP = forecasted pleasure; affective_att = affective attitudes. All scored were standardised (z-score).

*
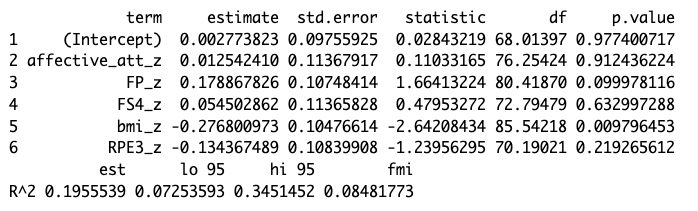
***FS4**

*Note.* FS4 = last affective response; bmi = body mass index; RPE3 = third perceived exertion score; RP = remembered pleasure; FP = forecasted pleasure; affective_att = affective attitudes. All scored were standardised (z-score).

**R outputs** **for Affective Responses 🡪 Forecasted Pleasure 🡪 Affective Attitudes 🡪 Daily total PA**

*
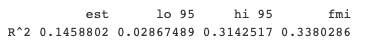
***
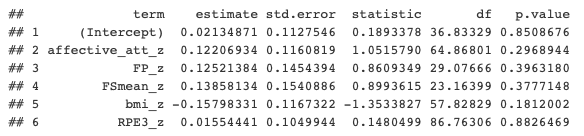
FSmean**

*Note.* FSmean = average affective response; bmi = body mass index; RPE3 = third perceived exertion score; RP = remembered pleasure; FP = forecasted pleasure; affective_att = affective attitudes. All scored were standardised (z-score).

**FS1**

## term estimate std.error statistic df p.value

## 1 (Intercept) -0.01347308 0.1043120 -0.1291614 50.73617 0.89774063

## 2 affective_att_z 0.09562611 0.1126272 0.8490501 68.89143 0.39879418

## 3 FP_z 0.19307221 0.1122098 1.7206365 70.94807 0.08967565

## 4 FS1_z -0.11756939 0.1062566 -1.1064662 64.95944 0.27260556

## 5 bmi_z -0.24250865 0.1140999 -2.1254066 59.65339 0.03770321

## 6 RPE3_z 0.06893454 0.1070561 0.6439104 70.00498 0.52173762

## est lo 95 hi 95 fmi

## R^2 0.1305191 0.02547153 0.2851584 0.242116

*Note.* FS1 = first affective response; bmi = body mass index; RPE3 = third perceived exertion score; RP = remembered pleasure; FP = forecasted pleasure; affective_att = affective attitudes. All scored were standardised (z-score).

**FS2**

## term estimate std.error statistic df p.value

## 1 (Intercept) -0.01089510 0.1037022 -0.1050615 53.53428 0.91671970

## 2 affective_att_z 0.09320561 0.1155001 0.8069741 62.02361 0.42276516

## 3 FP_z 0.15339063 0.1145688 1.3388514 70.77371 0.18490370

## 4 FS2_z 0.05182089 0.1221928 0.4240911 35.48791 0.67405916

## 5 bmi_z -0.21279157 0.1096336 -1.9409335 68.14109 0.05640402

## 6 RPE3_z 0.06013901 0.1075884 0.5589732 71.15678 0.57793502

## est lo 95 hi 95 fmi

## R^2 0.1235944 0.02454196 0.2705107 0.177811

*Note.* FS2 = second affective response; bmi = body mass index; RPE3 = third perceived exertion score; RP = remembered pleasure; FP = forecasted pleasure; affective_att = affective attitudes. All scored were standardised (z-score).

**FS3**

## term estimate std.error statistic df p.value

## 1 (Intercept) -0.01034678 0.1033924 -0.1000730 53.07364 0.9206635

## 2 affective_att_z 0.10193448 0.1144570 0.8905915 63.40426 0.3765154

## 3 FP_z 0.11285139 0.1260936 0.8949811 54.60932 0.3747251

## 4 FS3_z 0.12723932 0.1274461 0.9983779 44.26750 0.3235225

## 5 bmi_z -0.19310374 0.1119645 -1.7246866 65.63907 0.0892903

## 6 RPE3_z 0.07807947 0.1066714 0.7319627 76.82532 0.4664184

## est lo 95 hi 95 fmi

## R^2 0.1315889 0.03015738 0.2767265 0.1378179

*Note.* FS3 = third affective response; bmi = body mass index; RPE3 = third perceived exertion score; RP = remembered pleasure; FP = forecasted pleasure; affective_att = affective attitudes. All scored were standardised (z-score).

**FS4**

## term estimate std.error statistic df p.value

## 1 (Intercept) -0.01010781 0.1009541 -0.1001228 56.16026 0.92060340

## 2 affective_att_z 0.04083003 0.1136156 0.3593700 73.46135 0.72034925

## 3 FP_z 0.13087813 0.1107150 1.1821177 66.24733 0.24138476

## 4 FS4_z 0.22267778 0.1204123 1.8492941 53.32124 0.06996173

## 5 bmi_z -0.18278767 0.1095272 -1.6688790 66.01771 0.09987756

## 6 RPE3_z 0.10795764 0.1058311 1.0200939 79.65669 0.31077215

## est lo 95 hi 95 fmi

## R^2 0.1556912 0.04457871 0.3037872 0.1220033

*Note.* FS4 = last affective response; bmi = body mass index; RPE3 = third perceived exertion score; RP = remembered pleasure; FP = forecasted pleasure; affective_att = affective attitudes. All scored were standardised (z-score).

**R outputs** **for Affective Attitudes 🡪 Intentions**

**
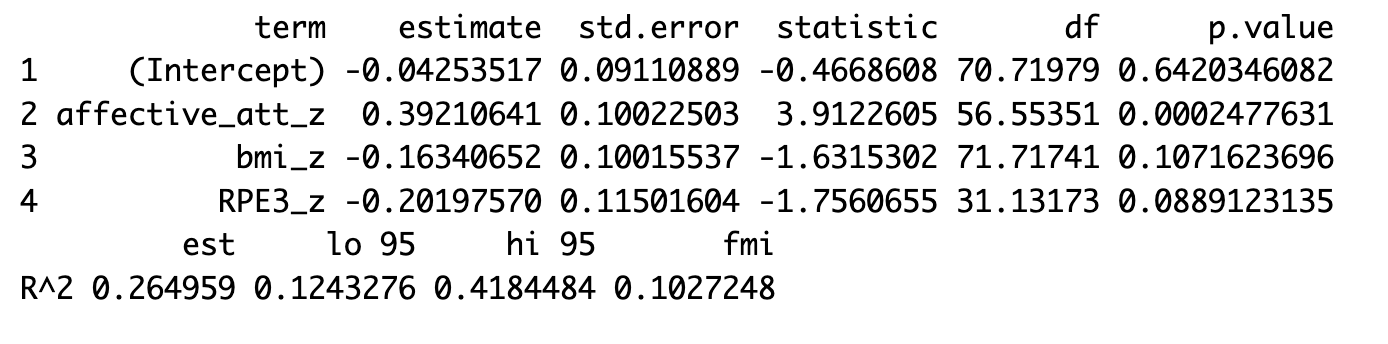
**

**R outputs** **for Affective Attitudes 🡪 Intentions 🡪 Daily MVPA**

term estimate std.error statistic df p.value

1 (Intercept) 0.01450555 0.1023900 0.1416697 66.79538 0.88776735

2 affective_att_z 0.03889991 0.1179729 0.3297359 62.57578 0.74270082

3 intention_nw_z 0.29803152 0.1142559 2.6084570 61.53065 0.01140109

est lo 95 hi 95 fmi

R^2 0.10007 0.0133042 0.242505 0.1979536

Coefficient StdError LowerCI UpperCI

1 0.01450555 0.1023900 -0.18987717 0.2188883

2 0.03889991 0.1179729 -0.19688147 0.2746813

3 0.29803152 0.1142559 0.06960263 0.5264604

**With confounders**

Regressions:

## Estimate Std.Err t-value df P(>|t|)

## affective_att_z ~

## FS4_z (a1) 0.347 0.134 2.587 468.374 0.010

## bmi_z (c1) -0.125 0.100 -1.242 Inf 0.215

## RPE3_z (c2) 0.122 0.113 1.077 Inf 0.281

## intention_nw_z ~

## affct__ (a2) 0.425 0.102 4.173 135.267 0.000

## FS4_z (a3) -0.104 0.115 -0.903 506.497 0.367

## bmi_z (c3) -0.181 0.106 -1.706 361.704 0.089

## RPE3_z (c4) -0.228 0.103 -2.208 73.736 0.030

## MVPA_acc_day_z ~

## intnt__ (b2) 0.205 0.116 1.770 107.583 0.080

## affct__ (b1) -0.014 0.116 -0.120 145.489 0.904

## FS4_z (c_pr) 0.108 0.121 0.887 310.887 0.376

## bmi_z (e1) -0.251 0.100 -2.525 296.215 0.012

## RPE3_z (e2) -0.081 0.101 -0.807 143.705 0.421

**R outputs** **for Affective Attitudes 🡪 Intentions 🡪 Daily total PA**

## term estimate std.error statistic df p.value

## 1 (Intercept) -0.006766458 0.1065576 -0.06350048 52.87029 0.9496075

## 2 affective_att_z 0.161522189 0.1196714 1.34971382 51.44269 0.1830176

## 3 intention_nw_z 0.116013775 0.1116329 1.03924389 64.82683 0.3025520

## est lo 95 hi 95 fmi

## R^2 0.05756114 0.001111298 0.1821935 0.1949205

**With confounders**

Regressions:

## Estimate Std.Err t-value df P(>|t|)

## affective_att_z ~

## FS4_z (a1) 0.352 0.140 2.517 935.472 0.012

## bmi_z (c1) -0.120 0.105 -1.148 178.013 0.253

## RPE3_z (c2) 0.107 0.118 0.905 271.801 0.366

## intention_nw_z ~

## affct__ (a2) 0.403 0.108 3.746 79.027 0.000

## FS4_z (a3) -0.098 0.122 -0.805 331.455 0.421

## bmi_z (c3) -0.183 0.109 -1.683 75.348 0.097

## RPE3_z (c4) -0.261 0.104 -2.504 72.662 0.015

## All_PA_acc_day_z ~

## intnt__ (b2) 0.103 0.135 0.760 168.821 0.449

## affct__ (b1) 0.047 0.119 0.391 124.287 0.697

## FS4_z (c_pr) 0.255 0.120 2.120 140.458 0.036

## bmi_z (e1) -0.171 0.103 -1.659 154.052 0.099

## RPE3_z (e2) 0.137 0.110 1.245 297.562 0.214

##

All scored were standardised (z-score).

**1. Self-Efficacy Pathway**

**R outputs** **for Affective Responses 🡪 Self-Efficacy Towards Exercise**

**
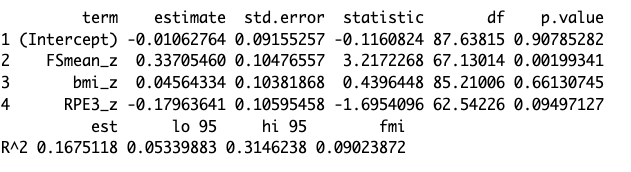
FSmean**

*Note.* FSmean = average affective response; bmi = body mass index; RPE3 = third perceived exertion score. All scored were standardised (z-score).

*
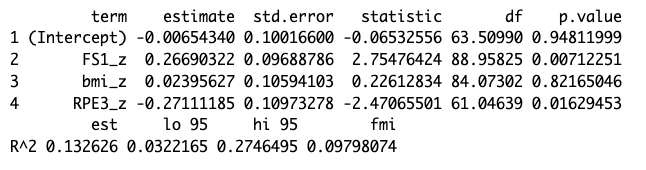
***FS1**

*Note.* FS1 = first affective response; bmi = body mass index; RPE3 = third perceived exertion score. All scored were standardised (z-score).

**
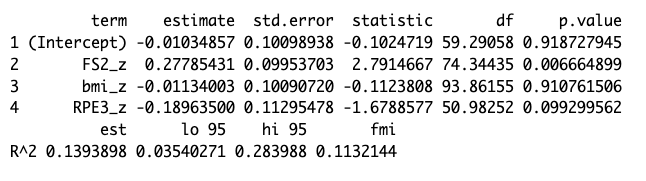
FS2**

*Note.* FS2 = second affective response; bmi = body mass index; RPE3 = third perceived exertion score. All scored were standardised (z-score).

*
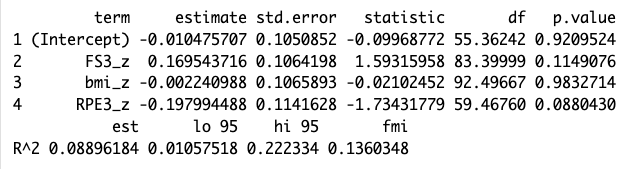
***FS3**

*Note.* FS3 = third affective response; bmi = body mass index; RPE3 = third perceived exertion score. All scored were standardised (z-score).

*
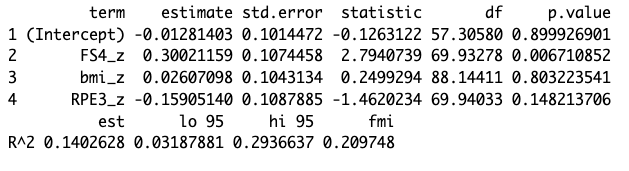
***FS4**

*Note.* FS4 = last affective response; bmi = body mass index; RPE3 = third perceived exertion score. All scored were standardised (z-score).

**R outputs** **for Self-Efficacy Towards Exercise 🡪 Self-Efficacy Towards Daily MVPA**

*
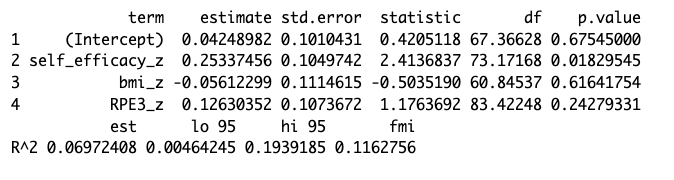
***FSmean**

*Note.* FSmean = average affective response; bmi = body mass index; RPE3 = third perceived exertion score; self_efficacy = self-efficacy towards exercise. All scored were standardised (z-score).

*
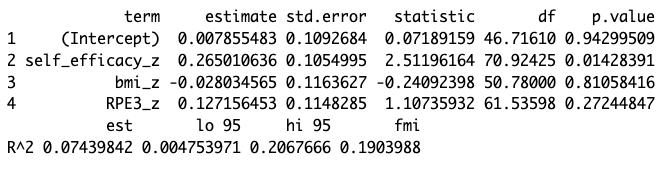
***FS1**

*Note.* FS1 = first affective response; bmi = body mass index; RPE3 = third perceived exertion score; self_efficacy = self-efficacy towards exercise. All scored were standardised (z-score).

*
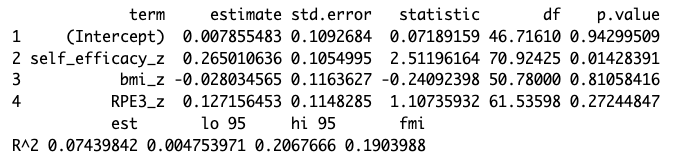
***FS2**

*Note.* FS2 = second affective response; bmi = body mass index; RPE3 = third perceived exertion score; self_efficacy = self-efficacy towards exercise. All scored were standardised (z-score).

*
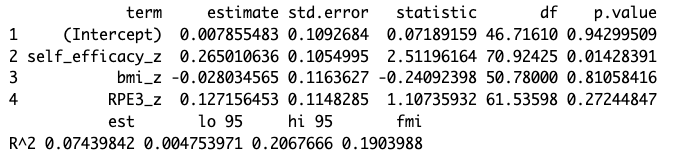
***FS3**

*Note.* FS3 = third affective response; bmi = body mass index; RPE3 = third perceived exertion score; self_efficacy = self-efficacy towards exercise. All scored were standardised (z-score).

*
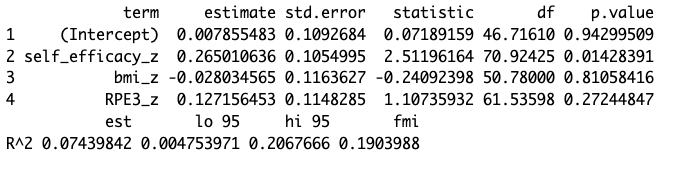
***FS4**

*Note.* FS4 = last affective response; bmi = body mass index; RPE3 = third perceived exertion score; self_efficacy = self-efficacy towards exercise. All scored were standardised (z-score).

**R outputs** **for Affective Responses 🡪 Self-Efficacy Towards Exercise 🡪 Self-Efficacy Towards Daily MVPA 🡪 Daily total PA**

**FSmean**

## term estimate std.error statistic df p.value

## 1 (Intercept) 0.026139841 0.1120814 0.23322187 35.30540 0.81693605

## 2 self_efficacyPA_z 0.266597989 0.1208179 2.20661039 39.40099 0.03323478

## 3 self_efficacy_z -0.014835980 0.1221577 -0.12144939 42.12579 0.90391258

## 4 FSmean_z 0.138678258 0.1300618 1.06624878 43.05006 0.29225464

## 5 bmi_z -0.182417569 0.1188146 -1.53531333 47.38490 0.13135768

## 6 RPE3_z -0.004216675 0.1063650 -0.03964346 85.88830 0.96846938

## est lo 95 hi 95 fmi

## R^2 0.1697045 0.04332881 0.3381045 0.3081655

*Note.* FSmean = average affective response; bmi = body mass index; RPE3 = third perceived exertion score; self_efficacy = self-efficacy towards exercise; self_efficacyPA = self-efficacy towards MVPA. All scored were standardised (z-score).

**FS1**

## term estimate std.error statistic df p.value

## 1 (Intercept) -0.008500265 0.1070258 -0.07942257 42.14249 0.937072470

## 2 self_efficacyPA_z 0.273174664 0.1028016 2.65730049 69.69467 0.009759333

## 3 self_efficacy_z 0.011750729 0.1079270 0.10887662 67.73582 0.913622252

## 4 FS1_z -0.114592457 0.1083627 -1.05749007 59.67004 0.294550452

## 5 bmi_z -0.280656356 0.1118246 -2.50979099 58.18733 0.014884087

## 6 RPE3_z 0.060985176 0.1149838 0.53038042 54.44569 0.598004864

## est lo 95 hi 95 fmi

## R^2 0.1469344 0.03795351 0.2964979 0.155252

*Note.* FS1 = first affective response; bmi = body mass index; RPE3 = third perceived exertion score; self_efficacy = self-efficacy towards exercise; self_efficacyPA = self-efficacy towards MVPA. All scored were standardised (z-score).

**FS2**

## term estimate std.error statistic df p.value

## 1 (Intercept) -0.007776686 0.1062973 -0.07315974 44.86104 0.94200424

## 2 self_efficacyPA_z 0.250650177 0.1092444 2.29439765 65.59270 0.02497708

## 3 self_efficacy_z -0.023904889 0.1070518 -0.22330211 74.50821 0.82391147

## 4 FS2_z 0.035370496 0.1203921 0.29379423 45.03922 0.77026480

## 5 bmi_z -0.249954729 0.1065978 -2.34484006 69.10071 0.02191421

## 6 RPE3_z 0.043557179 0.1128487 0.38597862 59.60610 0.70088676

## est lo 95 hi 95 fmi

## R^2 0.1374835 0.03273315 0.2851645 0.1534545

*Note.* FS2 = second affective response; bmi = body mass index; RPE3 = third perceived exertion score; self_efficacy = self-efficacy towards exercise; self_efficacyPA = self-efficacy towards MVPA. All scored were standardised (z-score).

**FS3**

## term estimate std.error statistic df p.value

## 1 (Intercept) -0.007315948 0.1048985 -0.06974312 45.66038 0.94470268

## 2 self_efficacyPA_z 0.238665054 0.1025470 2.32737135 71.40666 0.02278479

## 3 self_efficacy_z -0.031730863 0.1038102 -0.30566235 74.01209 0.76071981

## 4 FS3_z 0.149852357 0.1095072 1.36842499 61.51632 0.17615626

## 5 bmi_z -0.218367574 0.1086528 -2.00977356 67.01861 0.04848369

## 6 RPE3_z 0.067926281 0.1117636 0.60776746 62.49241 0.54554411

## est lo 95 hi 95 fmi

## R^2 0.1541573 0.04280634 0.3036506 0.141022

*Note.* FS3 = third affective response; bmi = body mass index; RPE3 = third perceived exertion score; self_efficacy = self-efficacy towards exercise; self_efficacyPA = self-efficacy towards MVPA. All scored were standardised (z-score).

**FS4**

## term estimate std.error statistic df p.value

## 1 (Intercept) -0.009463922 0.1016066 -0.09314279 51.29819 0.92615295

## 2 self_efficacyPA_z 0.205718641 0.1054794 1.95032124 65.70096 0.05540628

## 3 self_efficacy_z -0.061511082 0.1055890 -0.58255213 69.96446 0.56206764

## 4 FS4_z 0.224340132 0.1199929 1.86961121 53.03823 0.06706243

## 5 bmi_z -0.207135954 0.1084008 -1.91083421 62.81978 0.06059173

## 6 RPE3_z 0.085574801 0.1107732 0.77252279 64.14382 0.44264210

## est lo 95 hi 95 fmi

## R^2 0.172482 0.05659101 0.3202547 0.09079748

*Note.* FS4 = last affective response; bmi = body mass index; RPE3 = third perceived exertion score; self_efficacy = self-efficacy towards exercise; self_efficacyPA = self-efficacy towards MVPA. All scored were standardised (z-score).

**R outputs** **for Affective Responses 🡪 Self-Efficacy Towards Exercise 🡪 Self-Efficacy Towards Daily MVPA 🡪 Daily MVPA**

*
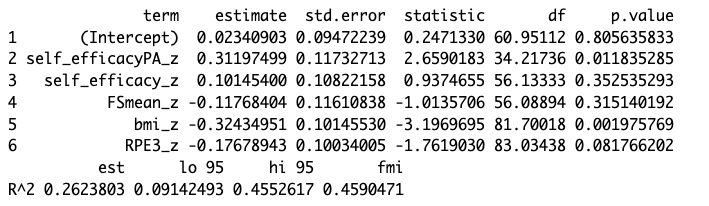
***FSmean**

*Note.* FSmean = average affective response; bmi = body mass index; RPE3 = third perceived exertion score; self_efficacy = self-efficacy towards exercise; self_efficacyPA = self-efficacy towards MVPA. All scored were standardised (z-score).

*
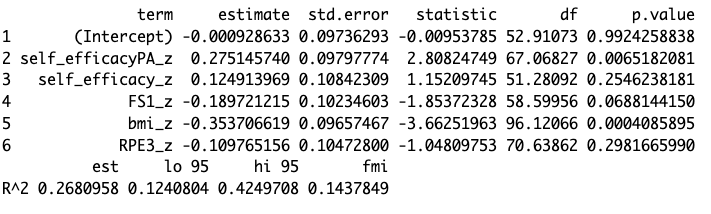
***FS1**

*Note.* FS1 = first affective response; bmi = body mass index; RPE3 = third perceived exertion score; self_efficacy = self-efficacy towards exercise; self_efficacyPA = self-efficacy towards MVPA. All scored were standardised (z-score).

*
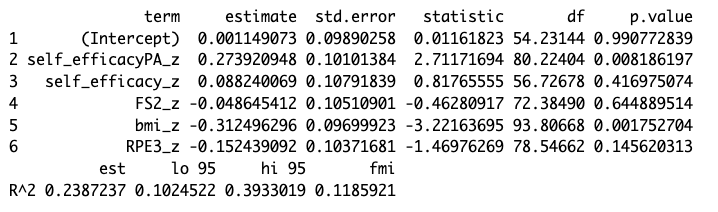
***FS2**

*Note.* FS2 = second affective response; bmi = body mass index; RPE3 = third perceived exertion score; self_efficacy = self-efficacy towards exercise; self_efficacyPA = self-efficacy towards MVPA. All scored were standardised (z-score).

*
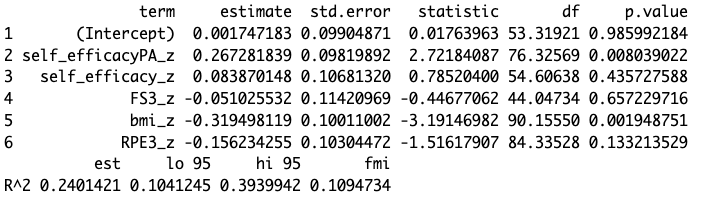
***FS3**

*Note.* FS3 = third affective response; bmi = body mass index; RPE3 = third perceived exertion score; self_efficacy = self-efficacy towards exercise; self_efficacyPA = self-efficacy towards MVPA. All scored were standardised (z-score).

*
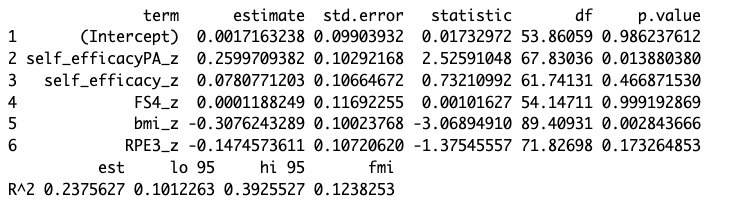
***FS4**

*Note.* FS4 = last affective response; bmi = body mass index; RPE3 = third perceived exertion score; self_efficacy = self-efficacy towards exercise; self_efficacyPA = self-efficacy towards MVPA. All scored were standardised (z-score).

**Table S3**

Multiple Mediation Effect Through Remembered Pleasure, Forecasted Pleasure and Affective Attitudes in the Association Between Affective Response and Subsequent Daily Time Spent in MVPA

| AR | Indirect effect via FP | | Indirect effect via AT | | Indirect effect (total) | | Total effect of AR on MVPA | |
| --- | --- | --- | --- | --- | --- | --- | --- | --- |
|  | *ß* | CI | *ß* | CI | *ß* | CI | *ß* | CI |
| FS_mean_ | .00 | -.00; .01 | .01 | -.04; .07 | .01 | -.04; .07 | -.06 | -.30; .20 |
| FS_1_ | .00 | -.01; .01 | -.001 | -.03; .02 | -.001 | -.03; .02 | -.17 | -.37; .03 |
| FS_2_ | .00 | -.01; .01 | .002 | -.03; .04 | .002 | -.03; .04 | .01 | -.17; .19 |
| FS_3_ | .00 | -.01; .01 | -.002 | -.04; .04 | -.001 | -.04; .04 | -.09 | -.34; .16 |
| FS_4_ | .00 | -.01; .01 | .003 | -.05; .09 | .003 | -.06; .09 | .06 | -.16; .28 |

*Note.* AR = affective response; FS = Feeling Scale; FP = forecasted pleasure; AT = affective attitudes; MVPA = moderate-to-vigorous physical activity; *ß* = standardised beta. FS was measured four times during an exercise session: at the start of the warm-up (FS_1_), at the start of the first exercises (FS_2_), at the start of the last exercises (FS_3_), and at the last minute of the session (FS_4_). FS_mean_ represents the mean score of the four FS scores. Remembered pleasure was not included in the complete model due to high correlation (r = .76) with forecasted pleasure. All association were controlled for body mass index and perceived exertion.

**Table S4**

Multiple Mediation Effect Through Self-Efficacy Towards Exercise and Towards MVPA in the Association Between the Last Affective Response and Subsequent Daily Time Spent in MVPA

| AR | Indirect effect via SE towards exercise | | Indirect effect via SE towards MVPA | | Indirect effect (total) | | Total effect of AR on MVPA | |
| --- | --- | --- | --- | --- | --- | --- | --- | --- |
|  | *ß* | CI | *ß* | CI | *ß* | CI | *ß* | CI |
| FS_mean_ | .03 | -.04; .14 | **.08** | **.01; .17** | **.11** | **.01; .25** | -.004 | -.23; .22 |
| FS_1_ | .03 | -.02; .12 | **.07** | **.01; .15** | **.11** | **.03; .21** | -.08 | -.28; .12 |
| FS_2_ | .03 | -.03; .11 | **.07** | **.01; .15** | **.10** | **.02; .21** | .05 | -.14; .23 |
| FS_3_ | .01 | -.02; .08 | **.07** | **.01; .15** | **.09** | **.01; .18** | .03 | -.20; .26 |
| FS_4_ | .02 | -.04; .12 | **.05** | **.002; .11** | .07 | -.004; .18 | .07 | -.16; .30 |

*Note.* AR = affective response; FS = Feeling Scale; FP = forecasted pleasure; SE = self-efficacy; MVPA = moderate-to-vigorous physical activity; *ß* = standardised beta. FS was measured four times during an exercise session: at the start of the warm-up (FS_1_), at the start of the first exercises (FS_2_), at the start of the last exercises (FS_3_), and at the last minute of the session (FS_4_). FS_mean_ represents the mean score of the four FS scores. All association were controlled for body mass index and perceived exertion. The coefficients in bold represent significant results (p < .05).

**Table S5**

Multiple Mediation Effect Through Remembered Pleasure, Forecasted Pleasure and Affective Attitudes in the Association Between Affective Response and Subsequent Daily Time Spent in total PA

| AR | Indirect effect via FP | | Indirect effect via AT | | Indirect effect (total) | | Total effect of AR on total PA | |
| --- | --- | --- | --- | --- | --- | --- | --- | --- |
|  | *ß* | CI | *ß* | CI | *ß* | CI | *ß* | CI |
| FS_mean_ | .00 | -.01; .02 | .01 | -.05; .08 | .01 | -.04; .09 | .15 | -.08; .39 |
| FS_1_ | .00 | -.01; .01 | -.00 | -.05; .02 | -.00 | -.05; .02 | -.12 | -.33; .09 |
| FS_2_ | .00 | -.01; .02 | .00 | -.03; .05 | .01 | -.03; .05 | .06 | -.14; .25 |
| FS_3_ | .00 | -.01; .20 | -.01 | -.08; .03 | -.01 | -.08; .03 | .12 | -.13; .36 |
| FS_4_ | .00 | -.01; .01 | .01 | -.05; .10 | .01 | -.05; .10 | **.23** | **.01; .46** |

*Note.* AR = affective response; FS = Feeling Scale; FP = forecasted pleasure; AT = affective attitudes; *ß* = standardised beta. FS was measured four times during an exercise session: at the start of the warm-up (FS_1_), at the start of the first exercises (FS_2_), at the start of the last exercises (FS_3_), and at the last minute of the session (FS_4_). FS_mean_ represents the mean score of the four FS scores. Remembered pleasure was not included in the complete model due to high correlation (r = .76) with forecasted pleasure. All association were controlled for body mass index and perceived exertion. The coefficients in bold represent significant results (p < .05).

**Table S6**

Multiple Mediation Effect Through Self-Efficacy Towards Exercise and Towards MVPA in the Association Between the Last Affective Response and Subsequent Daily Time Spent in total PA

| AR | Indirect effect via SE towards exercise | | Indirect effect via SE towards MVPA | | Indirect effect (total) | | Total effect of AR on total PA | |
| --- | --- | --- | --- | --- | --- | --- | --- | --- |
|  | *ß* | CI | *ß* | CI | *ß* | CI | *ß* | CI |
| FS_mean_ | -.01 | -.10; .09 | **.07** | **.01; .15** | .06 | -.04; .18 | .20 | -.02; .41 |
| FS_1_ | .00 | -.06; .07 | **.07** | **.01; .15** | .07 | -.01; .16 | -.04 | -.26; .17 |
| FS_2_ | -.01 | -.09; .08 | **.06** | **.00; .15** | .06 | -.03; .16 | .10 | -.11; .29 |
| FS_3_ | -.01 | -.06; .04 | **.06** | **.00; .14** | .05 | -.01; .14 | .20 | -.02; .43 |
| FS_4_ | -.02 | -.11; .06 | .04 | -.00; .10 | .02 | -.08; .11 | **.24** | **.02; .46** |

*Note.* AR = affective response; FS = Feeling Scale; FP = forecasted pleasure; SE = self-efficacy; *ß* = standardised beta. FS was measured four times during an exercise session: at the start of the warm-up (FS_1_), at the start of the first exercises (FS_2_), at the start of the last exercises (FS_3_), and at the last minute of the session (FS_4_). FS_mean_ represents the mean score of the four FS scores. All association were controlled for body mass index and perceived exertion. The coefficients in bold represent significant results (p < .05).

**Supplementary Material 7: Additional Descriptive Analyses**

**Figure S10**

*
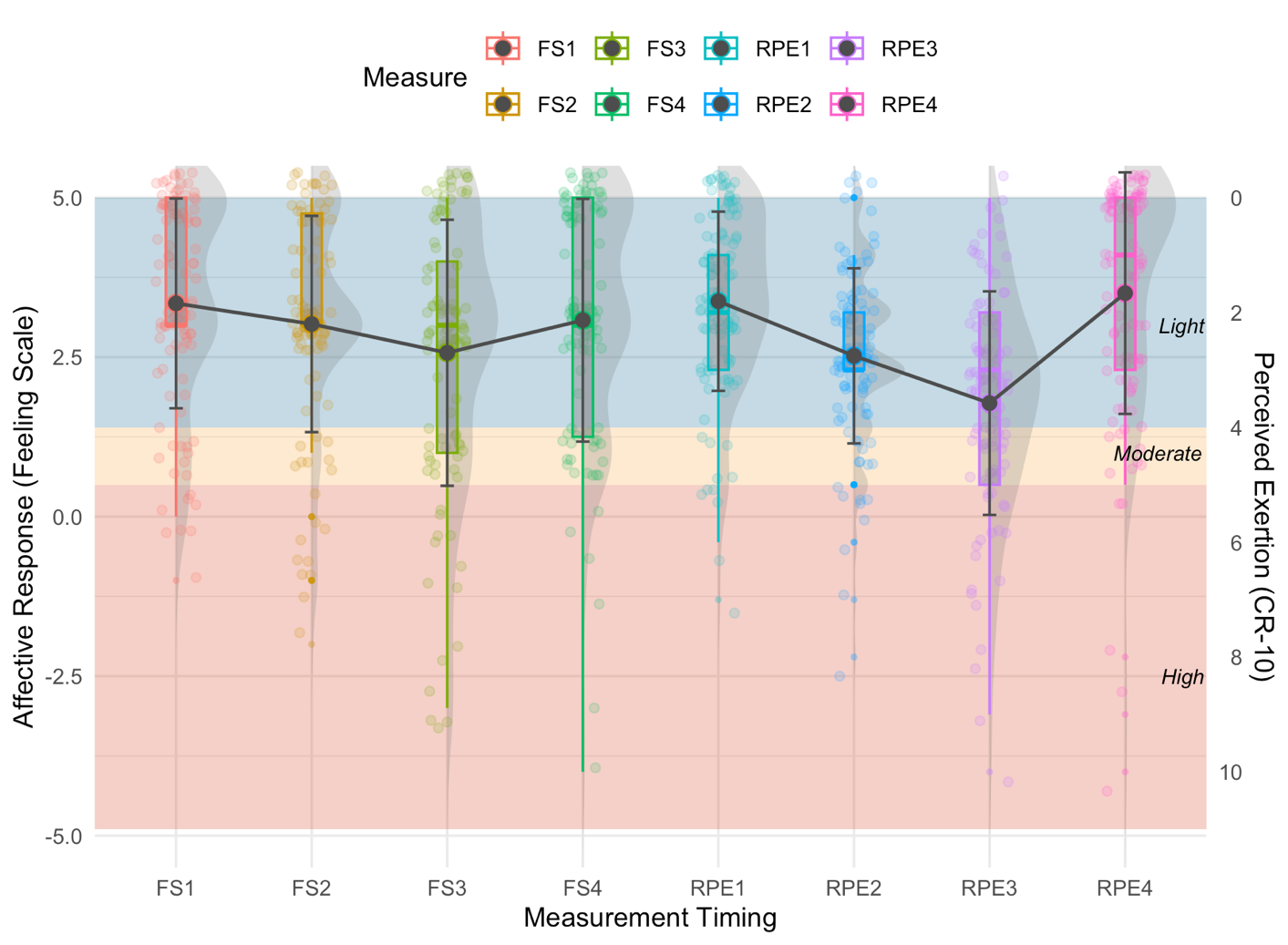
*Affective Responses and Perceived Exertion During the Exercise Session

*Note.* FS = feeling scale; RPE = rating of perceived exertion. FS and RPE were measured four times during an exercise session: at the start of the warm-up (FS_1_, RPE_1_), at the start of the first exercises (FS_2_, RPE_2_), at the start of the last exercises (FS_3_, RPE_3_), and at the last minute of the session (FS_4_, RPE_4_). Light intensity = RPE < 4, moderate intensity = RPE 4–5, high intensity = RPE > 5 (Bishop et al., 2025).

**References**

Bishop, D. J., Beck, B., Biddle, S. J. H., Denay, K. L., Ferri, A., Gibala, M. J., Headley, S., Jones, A. M., Jung, M., Lee, M. J. C., Moholdt, T., Newton, R. U., Nimphius, S., Pescatello, L. S., Saner, N. J., & Tzarimas, C. (2025). Physical Activity and Exercise Intensity Terminology: A Joint American College of Sports Medicine (ACSM) Expert Statement and Exercise and Sport Science Australia (ESSA) Consensus Statement. *Medicine & Science in Sports & Exercise*, *57*(11), 2599–2613. <https://doi.org/10.1249/MSS.0000000000003795>

Boiché, J., Gourlan, M., Trouilloud, D., & Sarrazin, P. (2019). Development and validation of the ‘Echelle de Motivation envers l’Activité Physique en contexte de Santé’: A motivation scale towards health-oriented physical activity in French. *Journal of Health Psychology*, *24*(3), 386–396. <https://doi.org/https://doi.org/10.1177/1359105316676626>

Cheval, B., Sarrazin, P., Isoard-Gautheur, S., Radel, R., & Friese, M. (2015). Reflective and impulsive processes explain (in) effectiveness of messages promoting physical activity: a randomized controlled trial. *Health Psychology*, *34*(1), 10–19. <https://doi.org/10.1037/hea0000102>

Cumming, G., & Finch, S. (2005). Inference by Eye: Confidence Intervals and How to Read Pictures of Data. *American Psychologist*, *60*(2), 170–180. <https://doi.org/10.1037/0003-066X.60.2.170>

Fessler, L., Maltagliati, S., Sieber, S., Cullati, S., Tessitore, E., Craviari, C., Luthy, C., Hanna, E., Meyer, P., Orsholits, D., Sarrazin, P., & Cheval, B. (2023). Physical activity matters for everyone’s health, but individuals with multimorbidity benefit more. *Preventive Medicine Reports*, *34*, e102265. <https://doi.org/10.1016/j.pmedr.2023.102265>

Foster, C., Florhaug, J. A., Franklin, J., Gottschall, L., Hrovatin, L. A., Parker, S., Doleshal, P., & Dodge, C. (2001). A new approach to monitoring exercise training. *The Journal of Strength & Conditioning Research*, *15*(1), 109–115.

Karpinski, A., & Steinman, R. B. (2006). The Single Category Implicit Association Test as a measure of implicit social cognition. *Journal of personality and social psychology*, *91*(1), 16–32. <https://doi.org/https://doi.org/10.1037/0022-3514.91.1.16>

Maltagliati, S. (2023). *The law of the least effort: identifying and manipulating automatic self-control processes in the context of physical activity* Université Grenoble Alpes [2020-..]]. <https://theses.fr/2023GRALS027>

Phipps, D. J., Hannan, T. E., Rhodes, R. E., & Hamilton, K. (2021). A dual-process model of affective and instrumental attitudes in predicting physical activity. *Psychology of Sport and Exercise*, *54*, e101899. <https://doi.org/10.1016/j.psychsport.2021.101899>
